# Sensori- and psychomotor abnormalities, psychopathological symptoms and functionality in schizophrenia-spectrum disorders: a network analytic approach

**DOI:** 10.1038/s41537-024-00547-0

**Published:** 2025-02-12

**Authors:** Stefan Fritze, Geva A. Brandt, Sebastian Volkmer, Jonas Daub, Dilsa Cemre Akkoc Altinok, Katharina M. Kubera, Christoph U. Correll, Georg Northoff, Andreas Meyer-Lindenberg, Dusan Hirjak

**Affiliations:** 1 Department of Psychiatry and Psychotherapy, Central Institute of Mental Health, Medical Faculty Mannheim, University of Heidelberg, Mannheim, Germany; 2 Hector Institute for Artificial Intelligence in Psychiatry, Central Institute of Mental Health, Medical Faculty Mannheim, Heidelberg University, Mannheim, Germany; 3 Center for Psychosocial Medicine, Department of General Psychiatry, University of Heidelberg, Heidelberg, Germany; 4 German Center for Mental Health (DZPG), Partner Site Berlin, Berlin, Germany; 5 Department of Child and Adolescent Psychiatry, Charité Universitätsmedizin Berlin, Berlin, Germany; 6 The Zucker Hillside Hospital, Department of Psychiatry, Northwell Health, Glen Oaks, NY USA; 7 Department of Psychiatry and Molecular Medicine, Donald and Barbara Zucker School of Medicine at Hofstra/Northwell, Hempstead, NY USA; 8 Mind, Brain Imaging and Neuroethics Research Unit, The Royal’s Institute of Mental Health Research, University of Ottawa, Ottawa, ON Canada; 9 German Centre for Mental Health (DZPG), Partner Site Mannheim-Heidelberg-Ulm, Mannheim, Germany

**Keywords:** Schizophrenia, Schizophrenia

## Abstract

Sensori- and psychomotor abnormalities are an inherent part of schizophrenia-spectrum disorders (SSD) pathophysiology and linked to psychopathological symptoms as well as cognitive and global functioning. However, how these different symptom clusters simultaneously interact with each other is still unclear. Here, we examined 192 SSD patients (37.75 ± 12.15 years, 73 females). First, we investigated the cross-sectional prevalence and overlap of individual sensori- and psychomotor abnormalities. Second, we applied network analysis methods to simultaneously model the associations between Neurological Soft Signs (NSS), level of akathisia, parkinsonism symptoms, tardive dyskinesia (TD) and catatonia signs as well as cognition, psychopathology, global functioning and daily antipsychotic dose. The largest centralities were exhibited by NSS (0.90), catatonia signs (0.82) and global functioning (0.79). NSS showed strong partial correlations with cognition and parkinsonism symptoms (edge weight, ew = 0.409 and ew = 0.318, respectively). Catatonia signs showed strong connections with global functioning (ew = 0.333). In contrast, TD, akathisia and daily antipsychotic dose were weakly connected with other variables (e.g., largest ew=0.176 between TD and akathisia). In conclusion, NSS and cognition, parkinsonism symptoms and NSS as well as catatonia signs and global functioning seem to be preferentially connected in SSD. The daily medication had little influence on sensori- and psychomotor abnormalities, indicating that they are features of core SSD pathophysiology. Future studies should incorporate these relationships to enhance the understanding of SSD.

## Introduction

In schizophrenia-spectrum disorders (SSD), sensorimotor abnormalities (such as neurological soft signs (NSS), tardive dyskinesia (TD), parkinsonism, and akathisia) and psychomotor phenomena (like catatonia signs) may present both in combination and in isolation, depending on the individual^1–3^. The RDoC matrix includes a sensorimotor domain but does not specifically address a psychomotor domain. As a result, we aim to differentiate these two clusters from both neurobiological and clinical perspectives, while acknowledging the overlapping nature of certain signs and symptoms. Understanding this complex interplay is crucial for elucidating the underlying neurobiological mechanisms, which may or may not be related to antidopaminergic medication treatment^1–7^. These signs and symptom clusters can be distinguished from both a neurobiological and a clinical perspective: From a neurobiological point of view, sensorimotor abnormalities are predominantly linked to dysfunctions in the primary motor areas, basal ganglia, and cerebellum^8,9^. In contrast, catatonia primarily manifests as psychomotor alterations, which are more closely associated with disruptions in the fronto-parietal (orbito- and prefrontal cortex) and limbic (e.g., amygdala and hypothalamus) regions^10,11^. From a clinical point of view, NSS encompass subtle deficits in motor coordination, sensory integration, and motor sequencing, reflecting early neurodevelopmental alterations^12–17^. TD, a condition characterized by involuntary repetitive movements, and parkinsonism, marked by psychomotor slowing, rigidity, and tremor, can result from prolonged antipsychotic use^18–20^ or spontaneously^21^. Similarly, akathisia, characterized by an inability to remain motionless and a subjective sense of inner restlessness, can be induced by acute antipsychotic use^6,22,23^. This association shows that antidopaminergic medication can modulate sensorimotor symptoms, although there are also findings suggesting that sensorimotor symptoms can develop without antidopaminergic medication^24–30^. Furthermore, sensorimotor disturbances, such as akathisia or parkinsonism signs, may exacerbate or mimic catatonic signs, complicating diagnostic assessment and treatment planning. Understanding how NSS, catatonia, akathisia, parkinsonism and TD intersect might help clinicians to differentiate between various sensori- and psychomotor disturbances commonly seen in SSD. This increased ability to differentiate sensorimotor and psychomotor abnormalities seems important since both sensori- and psychomotor abnormalities have been associated with worse treatment outcomes, including reduced symptom response to antidopaminergic medications, as well as worse social and cognitive functioning in SSD^21,31,32^.

A critical question concerns how sensori- and psychomotor abnormalities interact with cognitive function and overall global functioning. On one hand, these abnormalities may contribute to or exacerbate cognitive impairments through shared neural pathways, which could, in turn, lead to greater functional difficulties and a reduced quality of life^33^. On the other hand, sensori- and psychomotor disturbances can directly impact a person’s ability to initiate and engage in daily activities, participate in social interactions, use social gestures^34^, and maintain vocational performance, thereby affecting overall functional outcomes^33^.

Hitherto, studies have reported a plethora of cross-sectional and longitudinal associations between sensori- and psychomotor abnormalities and psychosocial functioning, global functioning, psychopathology, and cognitive functioning in SSD^1,21,31,32^^,35^^,36^. However, these studies have not been able to simultaneously take into account the dynamic relationship of different sensori- and psychomotor abnormalities with global functioning, psychopathology, cognitive functioning and daily antipsychotic dose. Studying the interplay between the symptom clusters mentioned above provides valuable insights into the complex presentation of SSD and may inform future treatment planning and intervention strategies aimed at maximizing cognitive and functional recovery and improving the quality of life of participants with SSD.

Therefore, this study had two main objectives: First, we sought to examine the prevalence of sensori- and psychomotor abnormalities in participants diagnosed with SSD, because this information might provide insights into the severity and nature of sensori- and psychomotor abnormalities, laying the groundwork for further investigations into the impact of sensori- and psychomotor abnormalities on cognitive and global functioning. Second, we used the network analytical approach^37,38^ to elucidate the complex interrelationships among different sensori- and psychomotor abnormalities. The unique strength of this network analytical approach is the ability to calculate pairwise partial correlations while simultaneously controlling for other variables and producing potent visualizations^37,38^. Thus, this network analytical approach allows to model, quantify and visualize the interdependent relationship between variables. We hypothesized that individual sensori- and psychomotor abnormalities would exhibit distinct patterns of associations with psychopathology, cognition, and global functioning. In particular, based on our previous studies^1,33^, we hypothesized that there would be a strong connection between NSS and parkinsonism as well as between NSS and cognition.

## Methods

### Study participants

In this study, we combined two independent cohorts of patients from different studies conducted at the Central Institute of Mental Health (CIMH).

*Cohort #1 (NSS study)*^1^ consisted of 129 subjects fulfilling the DSM-IV-TR^39^ criteria for SSD. This cohort has been used in previous studies of our group^1,40,41^. Diagnoses were made by staff psychiatrists and confirmed using the German versions of the Structured Clinical Interview for DSM-IV-TR axis I and II disorders (SCID) and examination of the case notes (SF and DH).

*Cohort #2 (whiteCAT* *study)*^42^ consisted of 63 subjects fulfilling the German Mini Diagnostic Interview for Mental Disorders (Mini-DIPS)^43^ criteria for SSD. Diagnoses were made by staff psychiatrists and confirmed using the examination of the case notes (GAB, JD, and DH). Patients from this cohort have already been used in other studies^44,45^.

Patients in all cohorts were excluded if they: (i) were aged <18 or >65 years; (ii) had a history of brain trauma or neurological disease (especially primary movement disorders); (iii) had alcohol/substance use disorder within 12 months prior to participation; or (iv) had MRI contraindications. The local Ethics Committees I and II (Medical Faculty Heidelberg and Medical Faculty Mannheim at Heidelberg University, Germany) approved the studies. We obtained written informed consent from all study participants after all aims and procedures of the study had been fully explained.

### Clinical assessment

Participants in both cohorts were examined during in- or outpatient treatment, typically after partial remission of acute psychopathological symptoms. All relevant study procedures (e.g., psychopathological rating scales, neuropsychological, sensori- and psychomotor assessments) were completed within 7 days.

In order to investigate NSS, we applied the Heidelberg NSS scale^46^. The Heidelberg NSS consists of five items assessing motor coordination (MOCO) [Ozeretski’s test, diadochokinesia, pronation/supination, finger-to-thumb opposition, speech articulation], three items assessing integrative functions (IF) [station and gait, tandem walking, two-point discrimination], two items assessing complex motor tasks [finger-to-nose test, fist-edge-palm test], four items assessing right/left and spatial orientation (RLSPO) [right/left orientation, graphesthesia, face-hand test, stereognosis], and two items assessing hard signs (HS) [arm holding test, mirror movements]. Ratings are given on a 0 (no prevalence) to 3 (marked prevalence) point scale. A sufficient internal reliability and test-retest reliability have been established previously^46,47^.

For the assessment of parkinsonism, we used the Simpson-Angus Scale (SAS)^48^. In particular, the SAS measures the presence and severity of ten symptoms, each rated on a scale from 0 to 4. A score of 0 means a normal motor function. A score of 1 indicates a visible motor abnormality. A score of 2 refers to a moderate motor abnormality. A score of 3 suggests marked motor symptoms. A score of 4 indicates severe motor abnormalities. In this study, SAS scoring was conservative, to avoid any ambiguities arising from e.g., limited cooperation or poor symptom reproducibility. If a symptom was not clearly present, then it was scored as absent (“0”). The SAS total score of all ten symptoms is a measure of global parkinsonism. The SAS also defines four major domains of parkinsonism, i.e., hypokinesia (1 item), rigidity (sum of 6 items), tremor (1 item), and glabella-salivation (2 items)^21^.

Investigating catatonia signs, the Northoff Catatonia Rating Scale (NCRS)^49^ measures the presence and severity of motor (13 items), affective (12 items), and behavioral (15 items) catatonia signs on a scale of 0 (not present) to 2 points (marked presence). Additionally, we applied ICD-11 criteria (https://icd.who.int/browse11) to investigate catatonia.

Examining akathisia, the Barnes Akathisia Rating Scale (BARS)^50^ uses the items objective, subjective (consisting of awareness and distress related to restlessness) and global clinical assessment on a scale of 0 (absence/no distress) to 3 points (marked or severe presence).

To identify patients with TD, we used the Abnormal Involuntary Movement Scale (AIMS)^51^ scores. The AIMS uses the items muscles of facial expression, lips and perioral, jaw, tongue, upper extremities, lower extremities, trunk, movements, severity of abnormal movements, incapacitation due to abnormal movements and patient´s awareness of abnormal movements on a scale of 0 (absence) to 4 points (severe).

The threshold value for NSS was a NSS total score of >7 (according to a mean value in healthy individuals^14,52^. A cutoff score of ≥4 on the total SAS score was employed to define parkinsonism based on previous studies addressing parkinsonism^21,53^. We used the NCRS criteria (score of ≥1 on each of the three subscales) for catatonia^54,55^ for the purpose of prevalence analysis, Venn Diagram and network analysis. In addition, anticipating the relevance of the recently introduced ICD-11 criteria of catatonia, we also employed these criteria for the purpose of the prevalence analysis and the Venn Diagram. The categorical definition of these criteria (rating options yes/no) unfortunately does not allow performing dimensional analyses as the NCRS does (rating options from 0 to 2 points). Also, NCRS comprises catatonia signs not present in ICD-11. Therefore, in order to depict the entire spectrum of catatonia signs (NCRS = 40 items vs. ICD-11 = 23 items), we performed the network analyses using the catatonia signs according to NCRS. The threshold value for akathisia was a BARS global score of ≥2^56^. Patients with TD were defined by the Schooler-Kane criteria^57,58^ based on items 1-7 of the AIMS. According to Schooler-Kane criteria^57,58^ a patient must have: (i) been exposed to antipsychotic medication for at least 3 cumulative months; (ii) no other conditions that could cause abnormal involuntary movements; and (iii) moderate dyskinetic movements in ≥1 body area (scoring ≥3 on AIMS) or mild dyskinetic movements in ≥2 body areas (scoring ≥2 on AIMS). For psychopathological assessments, patients were examined with the Positive and Negative Syndrome Scale (PANSS; including the positive, negative, and general subscores)^59^. Cognitive assessment was performed with the Brief Cognitive Assessment Tool for Schizophrenia (B-CATS)^60^ including Trail Making Test B (TMT-B), Digit Symbol Substitution Test (DSST) and Category Fluency (CF). Global functioning was investigated with the Global Assessment of Functioning (GAF)^61^ scale. Medical conditions potentially affecting central nervous system function, as well as cardiovascular or metabolic diseases in all study participants were excluded by physical examination, laboratory control, electrocardiography, electroencephalography, and magnetic resonance imaging (MRI).

Because not all patients with SSD fulfilled the established parkinsonism, catatonia, akathisia or TD criteria, we rather speak of *“parkinsonism symptoms”*, “catatonia signs”, *“akathisia symptoms”* and *“tardive dyskinesias”* (TDs), where appropriate throughout the manuscript.

### Statistical analyses

For statistical analyses, we used *R*, version 4.3.2 and *Rstudio*, version 2023.09.1^62^ (R Core Team, 2021). Initially, a descriptive analysis of demographic and clinical data of participants with SSD was performed.

In the first step, in a categorical approach using cut-off scores of the sensori- and psychomotor scales as described above, we investigated the prevalence and overlap of individual sensori- and psychomotor abnormalities. Here, we employed a Venn Diagram.

In a second step, we employed a dimensional approach with regard to the sensori- and psychomotor scales. The normality of the variables was investigated using histograms and skewness and kurtosis. We used the NSS total score, BARS global score, NCRS total score, SAS total score, AIMS total score, PANSS total score and GAF score. The B-CATS score was calculated by summing up z-scores of TMT-B, DSST and CF. Daily antipsychotic medication dose was converted to olanzapine equivalents (OLZe)^63^. In order to adjust for the confounding effects of age, sex and education, ordinary least squares regression was performed with age, sex and education as predictors and NSS total score, BARS global score, NCRS total score, SAS total score, AIMS total score, PANSS total score, GAF score, OLZe and B-CATS z-score as outcome variables (Supplementary Table 1) and the residuals were employed for network analysis^64,65^. In the network approach, nodes represent variables, and lines between variables are coined edges, representing relationships to be investigated. The most common psychological network estimation technique (pairwise Markov random fields [PMRF]) uses partial correlations (edge weights) between variables (nodes), which are calculated after controlling for all other variable correlations. Here, green edges represent positive correlations, while red edges represent negative correlations. Thicker edges refer to stronger correlations, while thinner edges refer to weaker correlations. This description refers to Fig. 2, although there were no negative correlations and thus no red edge in the results. Due to the violation of normality in our variable distributions, we used a non-paranormal transformation in preparing the data as follows: employing the bootnet package^66^, we set the default option of the estimateNetwork function to “huge”. Network estimation involved a graphical Gaussian model (GGM)^64,66^. GGM assumes data in a normal distribution. Non-paranormal transformation is a method to transform continuous data which are not normally distributed, leading to normal distributions. We employed this method because our variables were not normally distributed.

**Table 1:** Overview of performed analyses.

Technique	Network analysis	Network analysis	Network analysis	Network analysis	Network analysis	Network analysis	Wilcoxon rank sum test/ Chi-square test	Spearman correlation	Network analysis
Included variables	Five sensori- and psychomotor nodes, PANSS total score, GAF score, B-CATS, OLZe after regressing out age, sex and education	Five sensori- and psychomotor nodes, PANSS total score, GAF score, B-CATS, OLZe	Five sensori- and psychomotor nodes after regressing out age, sex and education	Five motor nodes	Five sensori- and psychomotor nodes, PANSS total score, GAF score, B-CATS, OLZe after regressing out age, sex, and education, focusing on nodewise predictability	Five sensori- and psychomotor nodes, PANSS total score, GAF score, B-CATS, OLZe after regressing out age, sex and education, focusing on betweenness/ closeness	Comparison of clinical and demographic variables: Cohort #1 (*n* = 129) vs. cohort #2 (*n* = 63)	Spearman correlations of B-CATS with sensori- and pychomotor nodes	Five sensori- and psychomotor nodes, PANSS subscores (Positive, Negative, General), GAF score, B-CATS, OLZe after regressing out age, sex and education
Stability	Stable	Stable	EI stable; strength unstable	EI stable; strength unstable	Stable	Unstable	-	-	Stable

*AIMS* Abnormal Involuntary Movement Scale, *BARS* Barnes Akathisia Rating Scale, *B-CATS* Brief Cognitive Assessment Tool for Schizophrenia, *GAF* Global Assessment of Functioning Scale, *NSS* Neurological Soft Signs, *OLZe* Olanzapine equivalents, *PANSS* Positive and Negative Syndrome Scale, *SAS* Simpson Angus Scale, *SSD* Schizophrenia Spectrum Disorders.

Five sensori- and psychomotor nodes: NSS total score, BARS global score, NCRS total score, SAS total score, AIMS total score.

We applied the extended Bayesian information criterion (EBIC)^66,67^. To avoid false-positive relationships between nodes, we regularized the network with the least absolute shrinkage and selection operator (LASSO)^66,67^. LASSO is a type of regularization, i.e., when estimating network parameters, the fit of a model is penalized by how many edges are included in the network. When using LASSO, some edge-weights are shrinked and estimated to equal exactly zero. Thus, a sparse model results, reducing the probability of false-positive edges. LASSO regularization is a well-established technique in cases with few observations and many variables. The qgraph package^68^ and Fruchterman-Reingold-algorithm were used for visualization^67^. This algorithm places nodes with stronger correlations more centrally. However, visual interpretation of these two-dimensional representations of three-dimensional structures should be done with caution. In order to understand the estimated network structure, the assessment of node centrality and edges is important^38,66,67^. To assess node centrality, expected influence (EI) and strength were calculated. EI of a node represents the sum of all partial correlations (accounting for negative edges) and indicates its importance within a network graph. Higher values indicate stronger interconnectedness. Strength is the sum of absolute edge weights. To examine EI and strength stability, we employed a bootstrap approach with the bootnet package. We also calculated edge weights (partial correlation coefficients between all network nodes) and performed edge stability analyses. Further, we investigated whether network node centrality differed significantly between nodes and whether network edge weights differed significantly between edges.

For completeness, we conducted a network analysis including five sensori- and psychomotor nodes, PANSS total score, GAF score, B-CATS, OLZe without regressing out age, sex, and education (s. Table 1).

Further, to examine the general structure and interconnections of sensori- and psychomotor processes in isolation, providing a foundational understanding of how these processes relate to each other, we also conducted a network analysis focusing exclusively on the sensori- and psychomotor nodes both with and without regressing out the effects of age, sex, and education (Supplementary Table 1). By analyzing this subset independently, we aimed to establish a baseline network before incorporating additional variables such as medication, functioning, cognition, and psychopathology into the analysis.

Finally, we conducted an additional analysis of nodewise predictability to examine how well a given node can be predicted by all other nodes it is connected to in the network^69^.

## Results

### Clinical and demographic characteristics

**Fig. 1:**
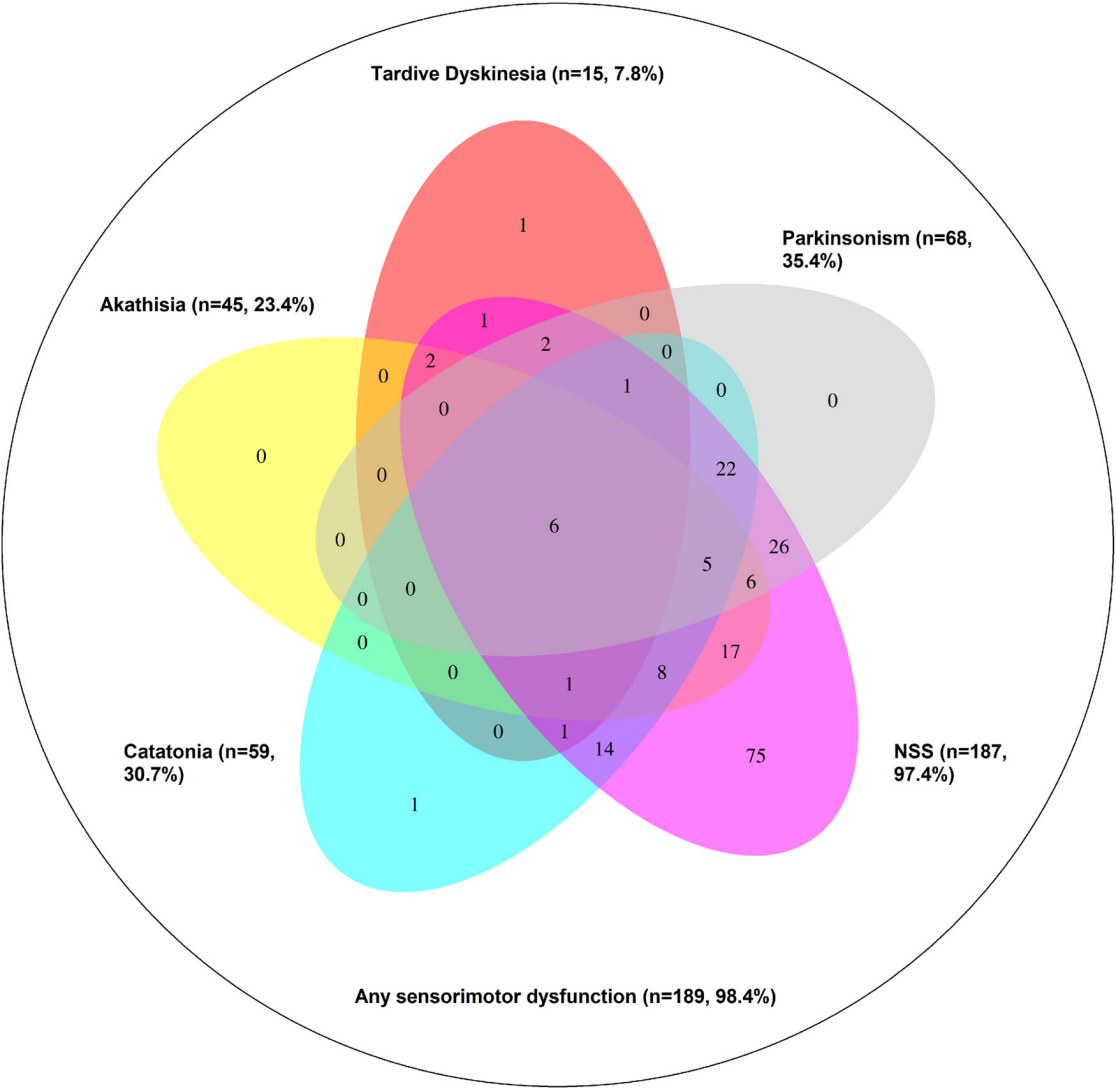
Venn diagram showing the prevalence and overlap of sensori- and psychomotor abnormalities in SSD patients (n = 192). The diagram illustrates that neurological soft signs (NSS) are highly prevalent and often occur independently, while other abnormalities such as tardive dyskinesia, akathisia, parkinsonism, and catatonia show significant overlap with NSS but are rarely present in isolation. The size and overlap of each ellipse reflect the frequency and co-occurrence of these abnormalities. For instance, n = 75 participants fulfilled NSS cut-off criteria without fulfilling cut-off criteria of any other sensori- or psychomotor abnormality. Further, *n* = 17 participants fulfilled cut-off criteria of NSS and akathisia, without fulfilling cut-off criteria of any other sensori- or psychomotor abnormality.

Demographic and clinical characteristics of study participants and the included variables as well as skewness and kurtosis for each variable are shown in Table 2. The prevalences of categorically defined sensori- or psychomotor symptoms/signs were as follows: NSS: 187 (97.4%), parkinsonism: 68 (35.4%), catatonia (according to NCRS): 59 (30.7%), akathisia: 59 (30.7%), and TD: 15 (7.8%) (Fig. 1). The largest overlaps (i.e., simultaneously fulfilled cut-off criteria) between sensorimotor categories were found among NSS and SAS (*n* = 68, 35.4%, Fig. 1), NSS and NCRS (*n* = 59, 30.7%, Fig. 1), NSS and BARS (*n* = 45, 23.4%, Fig. 1) and NCRS and SAS (*n* = 34, 17.71%, Fig. 1). Of the 192 included participants, 189 (98.4%) exhibited at least one sensori- or psychomotor symptom/sign. Additionally, we performed this analysis with ICD-11 criteria for catatonia. Here, only 39 participants fulfilled criteria for catatonia and consequently smaller overlaps between sensori- and psychomotor dysfunction categories occurred, yet the patterns of overlap remained similar (supplementary fig. 6).

**Table 2:** Clinical and demographic variables in schizophrenia spectrum disorders (SSD; *n* = 192).

Variable	Mean ± SD	Skewness	Kurtosis
Age (years)	37.75 ± 12.15	0.25	2.00
Sex (male/female)	119/73	-	-
Education (years)	13.22 ± 2.98	0.31	4.24
OLZe	16.18 ± 10.33	0.61	3.28
PANSS total score	67.02 ± 19.64	0.53	3.27
GAF score^a^	58.25 ± 19.25	0.03	2.06
NSS total score	20.45 ± 8.89	0.82	3.26
NCRS total score	4.08 ± 4.14	1.47	6.34
SAS total score	3.29 ± 2.86	1.32	4.77
AIMS total score	1.09 ± 2.39	2.90	11.35
BARS global score	0.78 ± 1.19	1.52	4.59
TMT-B	111.22 ± 65.14	1.64	5.65
DSST^a^	63.15 ± 19.33	−0.003	3.28
CF^a^	15.76 ± 4.50	−0.41	2.99

*SD* Standard Deviation*, PANSS* Positive and Negative Symptoms Scale, *GAF* Global Assessment of Functioning Scale, *NSS* Neurological Soft Signs, *NCRS* Northoff Catatonia Rating Scale, *SAS* Simpson Angus Scale, *AIMS* Abnormal Involuntary Movement Scale, *BARS* Barnes Akathisia Rating Scale, *TMT-B* Trail Making Test B, *DSST* Digit Symbol Substitution Test, *CF* Category Fluency.

^a^Reverse-coded values

### Network analysis

**Fig. 2:**
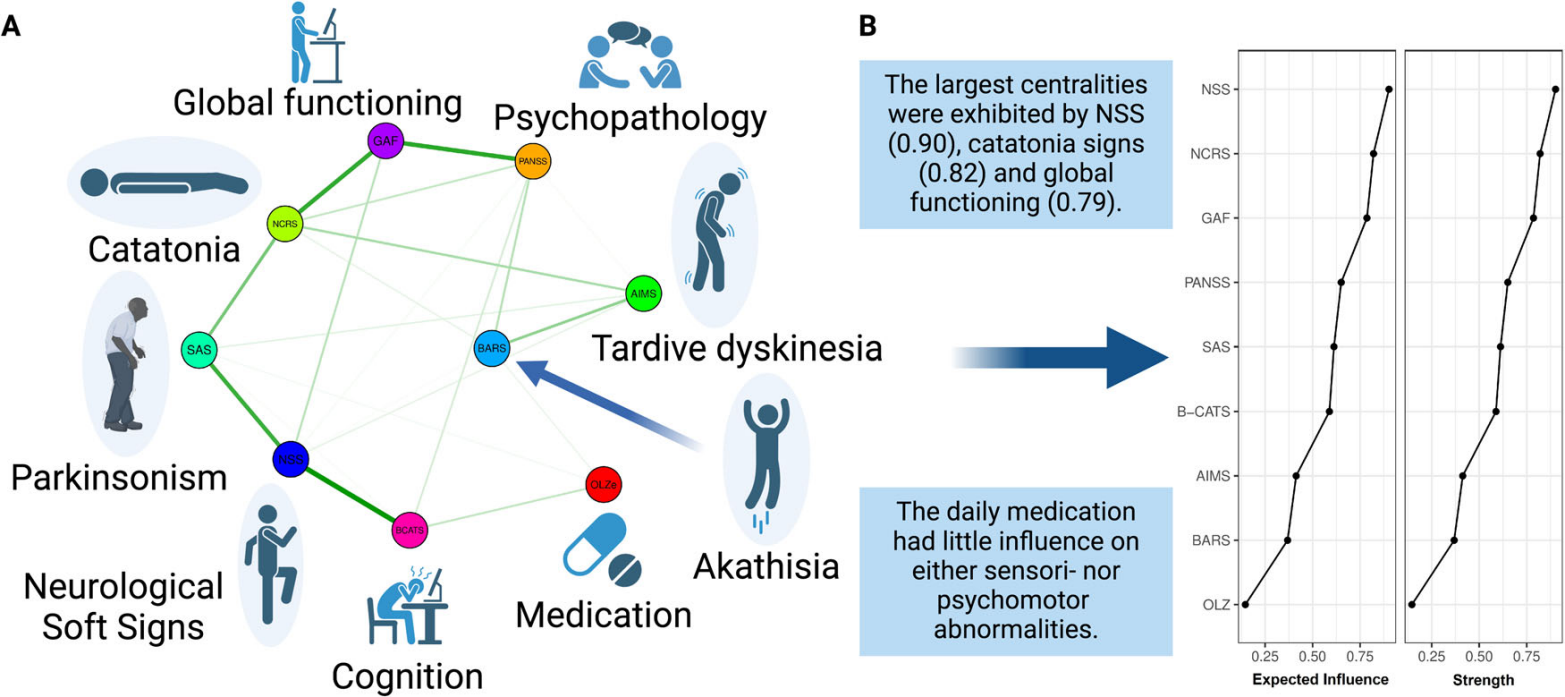
Study workflow showing the respective clinical rating scales and results of the network analysis as well as the centrality of each node in SSD patients (*n* = 192). **A** shows that NSS were closely connected to parkinsonism and cognition. Catatonia signs were closely connected to global functioning. Antipsychotic medication showed weak associations with other network nodes. Green edges refer to positive relationships. **B** shows that the largest centralities were exhibited by NSS, catatonia signs and global functioning.

The network included the NSS total, BARS global, NCRS total, SAS total, AIMS total, PANSS total, GAF and B-CATS z-scores as well as daily OLZe doses (Fig. 2A). Network edges as well as centrality (expected influence [EI] and strength) were deemed stable (Supplementary Figs. 1 and 2).

The largest centralities were exhibited by NSS (0.90), catatonia signs (0.82), and global functioning (0.79) (Fig. 2B). The network’s largest centrality, exhibited by NSS, was significantly larger than centralities of B-CATS, AIMS, BARS and OLZe, yet not significantly larger than centralities of NCRS, GAF, PANSS and SAS (Fig. 2B and Supplementary Figs. 3 and 4).

The networks’ strongest partial correlation (edge weight) was identified between NSS and B-CATS (ew=0.409, Supplementary Table 3). This partial correlation was significantly stronger than the network’s remaining partial correlations, except for the edge weights NCRS-GAF, PANSS-GAF, and NSS-SAS (Table 3 and Supplementary materials Fig. 5). The strongest connection of the SAS arose with NSS (ew = 0.318, Supplementary Table 2). The strongest connection of NCRS arose with GAF (ew = 0.333, Supplementary Table 3). The strongest, still relatively weak, connection of AIMS and BARS arose between each other (ew = 0.176, Supplementary Table 3). Both showed weak connections with other variables (Table 3). Daily OLZe doses showed weak connections with all other variables (Table 3). The connection of daily OLZe doses with B-CATS was numerically (albeit not significantly) larger than its connection with any sensorimotor dysfunction category (Table 3).

**Table 3:** Partial correlation coefficients (edge weights) according to network analysis.

	OLZe	PANSS	NCRS	AIMS	SAS	BARS	NSS	GAF	B-CATS
**OLZe**	0	0	0	0	0.019	0.030	0	0	0.097
**PANSS**	0	0	0.094	0.013	0	**0.113**	0.016	**0.345**	0.069
**NCRS**	0	0.094	0	**0.138**	**0.218**	0.038	0	**0.333**	0
**AIMS**	0	0.013	**0.138**	0	0.046	**0.176**	0.041	0	0
**SAS**	0.019	0	**0.218**	0.046	0	0	**0.318**	0	0.013
**BARS**	0.030	**0.113**	0.038	**0.176**	0	0	0.010	0	0
**NSS**	0	0.016	0	0.041	**0.318**	0.010	0	**0.108**	**0.409**
**GAF**	0	**0.345**	**0.333**	0	0	0	**0.108**	0	0
**B-CATS**	0.098	0.069	0	0	0.013	0	**0.409**	0	0

Partial correlations between variables. Higher values indicate stronger associations between variables after controlling for all other variables. Bold partial correlations are >0.100. For instance, the partial correlation coefficient between PANSS and GAF is 0.345, after controlling for all other variables. The table does not specify whether the associations are significantly different. Please refer to Supplementary Fig. 5 (edge-weight difference test) to check whether the presented associations are significantly different.

*PANSS* Positive and Negative Symptoms Scale, *NCRS* Northoff Catatonia Rating Scale, *AIMS* Abnormal Involuntary Movement Scale, *SAS* Simpson Angus Scale, *BARS* Barnes Akathisia Rating Scale, *B-CATS* Brief Cognitive Assessment Tool for Schizophrenia, *NSS* Neurological Soft Signs, *GAF* Global Assessment of Functioning Scale.

In addition, the network analysis including five sensori- and psychomotor nodes, PANSS total score, GAF score, B-CATS, OLZe without regressing out age, sex and education yielded a pattern similar to our results after regressing out age, sex and education. Here, the largest centralities were also exhibited by NSS, GAF, and NCRS (see supplementary Fig. 7).

Further, concerning edge weights, the resulting networks focusing solely on sensorimotor and psychomotor nodes showed the following similarities with our network analyses including also PANSS total score, OLZe, GAF score, and B-CATS. In the networks focusing solely on sensorimotor and psychomotor nodes the connection between NSS and SAS remained strong and the connection between AIMS and BARS remained stronger than the connection of AIMS and BARS with other nodes. Concerning centrality, first, after adjusting for age, sex, and education (see Supplementary Figs. 11 and 12), the centrality of expected influence for SAS (0.68) and NCRS (0.66) was nominally higher than for NSS (0.64). However, these differences were not statistically significant based on the centrality difference test (see supplementary fig. 13). Notably, expected influence emerged as the only stable centrality parameter across these conditions. Second, without adjusting for age, sex, and education (see Supplementary Fig. 14), the centrality values of SAS (0.72), AIMS (0.70), and NCRS (0.60) were nominally higher than NSS (0.57), but again, these differences were not significant according to the centrality difference test. In both analytical approaches—whether controlling for demographic variables or not—BARS consistently demonstrated significantly lower centrality compared to SAS (see supplementary materials).

**Fig. 3:**
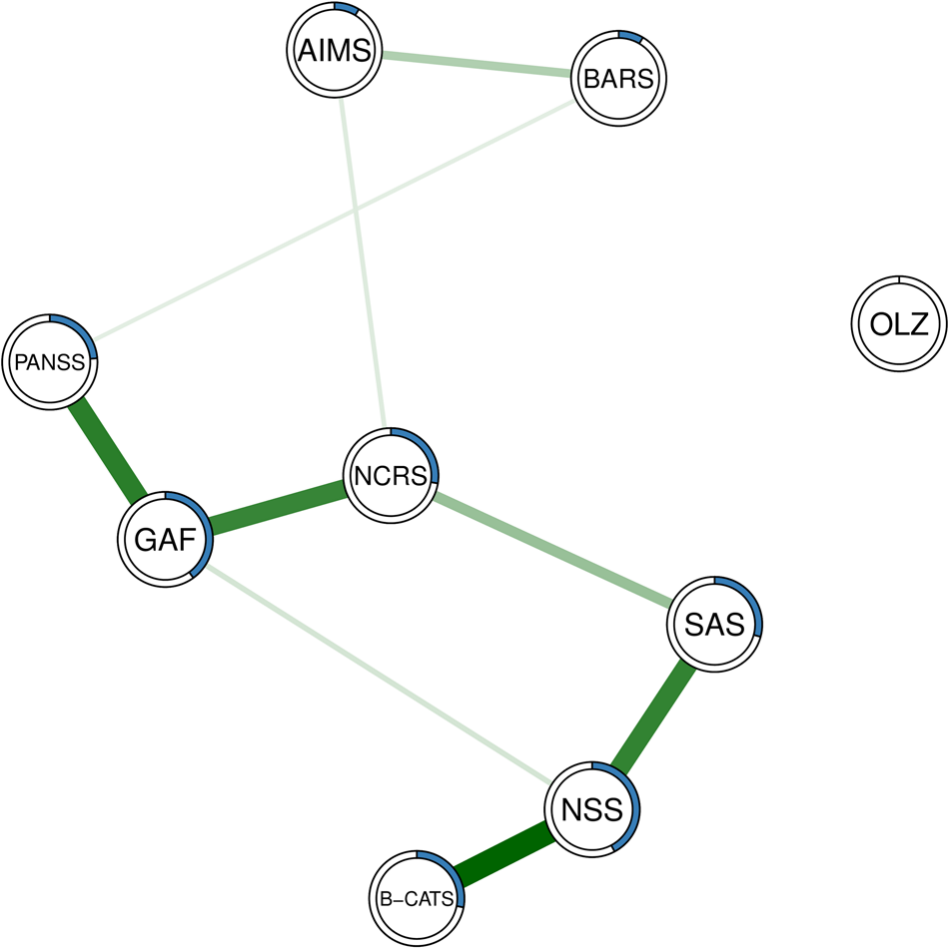
Nodewise predictability estimated on sensori- and psychomotor nodes, PANSS total score, GAF score, B-CATS, OLZe after regressing out age, sex and education. Green edges refer to positive relationships. Blue rings indicate the proportion of explained variance (*R*²).

Finally, consistent with our centrality analyses, NSS (*R*² = 0.422) and GAF (*R*² = 0.400) exhibited the highest nodewise predictability (s. Table 4 and Fig. 3). Across our network, nodewise predictability ranged from *R*² = 0 to *R*² = 0.422, which we interpret as indicating low to moderate predictability, suggesting that unmeasured variables play a significant role in shaping the relationships within the network.

**Table 4:** The percentage of explained variance for each of the variables (*R*²) calculated using nodewise predictability.

Variable	*R*²
OLZe	0.000
BARS_global_	0.085
AIMS_total score_	0.086
PANSS_total score_	0.237
NCRS_total score_	0.276
B-CATS	0.280
SAS_total score_	0.292
GAF	0.400
NSS_total score_	0.422

*PANSS* Positive and Negative Symptoms Scale, *NCRS* Northoff Catatonia Rating Scale, *AIMS* Abnormal Involuntary Movement Scale, *SAS* Simpson Angus Scale, *BARS* Barnes Akathisia Rating Scale, *B-CATS* Brief Cognitive Assessment Tool for Schizophrenia, *NSS* Neurological Soft Signs, *GAF* Global Assessment of Functioning Scale.

## Discussion

This study represents the first cross-sectional modeling and quantification of the complex network structure of the most prevalent sensori- and psychomotor abnormalities in SSD. This analysis was conducted while simultaneously controlling for key factors such as cognition, psychopathology, global functioning, and daily antipsychotic medication dosage. Centrality, defined as the sum of partial correlations associated with a specific node, serves as a critical metric for understanding the importance and influence of individual nodes within the network. In network analysis, nodes represent structural elements corresponding to variables of interest, while edges signify the connections between them. Investigating both nodes and edges is essential to derive comprehensive and meaningful insights from the network structure. Five main findings emerged: First, the categorical analysis of prevalences yielded significant overlap among NSS, parkinsonism symptoms, akathisia symptoms, TDs, and catatonia signs, reflecting the clinical complexity and the scientific challenges when investigating sensori- and psychomotor abnormalities in SSD. Second, network analysis showed that NSS, catatonia signs, and global functioning emerged as nodes with the largest centralities. Third, NSS was strongly associated with cognition and parkinsonism symptoms. Fourth, catatonia signs showed strong connections with global functioning. Finally, daily antipsychotic medication dose showed low centrality and weak connections with all sensori- and psychomotor abnormalities.

First, we found a notable overlap among NSS, parkinsonism symptoms, akathisia symptoms, TDs, and catatonia signs. The phenomenon of symptom co-occurrence (across several mental disorders) has also been observed in other studies^70,71^, but the co-occurrence of sensori- and psychomotor abnormalities across SSD has barely been investigated. In the present study, the vast majority of patients met the cut-off criteria for at least one sensori- or psychomotor category and even more showed subthreshold signs and symptoms. This partial symptom presentation reflects the clinical reality that sensori- and psychomotor abnormalities often do not present in a neatly categorized or fully developed form, but rather on a continuum. We can only speculate about the reasons, perhaps this large presence and overlap of sensori- and psychomotor abnormalities in people with SSD is due to the individual clinical rating scales, which tend to depict clinically observable constructs rather than neurobiological mechanism underlying the sensori- and psychomotor abnormalities. That said, the prevalences of sensori- and psychomotor abnormalities in our study continue to be higher than those published by Peralta et al. ^72^, which were 35.4% vs 21% for parkinsonism, 30.7% vs 19% for catatonia, 23.4% vs 4% for akathisia and 7.8% vs 9% for TD. The prevalence of NSS was not reported by Peralta et al. ^72^. This difference in prevalence could be due to different measured and unmeasured sample characteristics (for example, participants in our sample were not antipsychotic-naïve and older). Also, we applied a relatively low cut-off criterion to define NSS. In line with this, the centrality of NSS in our analysis could partly reflect the high prevalence of NSS due to the measure’s high sensitivity and relatively low specificity. However, it is important to note that NSS are thought to have a neurodevelopmental origin and are also commonly observed in healthy individuals, individuals at ultra-high risk (UHR), and relatives of patients with schizophrenia spectrum disorders^14,73^. This broader presence suggests that NSS may represent a foundational or vulnerability marker for the development of other sensori- and psychomotor abnormalities. As a consequence of the higher prevalence of sensori- and psychomotor abnormalities, overlaps (defined as participants fulfilling criteria of more than one sensori- and psychomotor abnormality) occur more frequently. In addition, Walther et al. ^36^ discussed whether the co-occurrence of motor signs may be attributable to a lack of conceptual clarity, e.g., when considering overlapping definitions and distinct vocabulary for similar phenomena such as stupor, bradykinesia or motor retardation. Also, some items of sensori- and psychomotor scales show overlap with items of other scales, e.g., rigor occurs in catatonia and parkinsonism rating scales. Difficulties when walking are present in both the Heidelberg NSS scale and the SAS scale. However, studies on co-occurrence and overlap between different categories of sensori- and psychomotor abnormalities, particularly in relation to neurobiology, are lacking. Previous MRI studies showed that similar sensori- and psychomotor-related regions (e.g., orbito- and prefrontal cortex, primary motor area, basal ganglia, and thalamus) are involved in NSS^12,74^ as well as in catatonia^10,11^ and parkinsonism^75,76^.

Second, since centrality reflects one node’s connections with all other nodes, the strong connections of NSS, catatonia signs, and global functioning with all other nodes suggest their critical roles in the network structure in SSD. Notably, the centrality of NSS was significantly larger than the centralities of akathisia, tardive dyskinesia, cognition, and medication, suggesting a hierarchy of individual domain connections within the network. The ubiquity of NSS in different cohorts, including healthy individuals^14,52^ and those at increased risk of psychosis^77^ due to genetic, environmental, or other predisposing factors, emphasizes the importance of NSS as markers of underlying neurobiological vulnerabilities rather than as direct indicators of specific pathological abnormalities in the brain. Furthermore, NSS are indicators of sensorimotor dysfunction vulnerability in SSD. The high centrality of NSS in the present study confirmed and extended previous study results by Fritze et al. ^33^ in a transdiagnostic sample of 38 patients with mood disorders and 172 patients with SSD, focusing on NSS *subscores* while excluding akathisia, parkinsonism, catatonia, and TD. The present study sample and the SSD sample investigated by Fritze et al. ^33^ were not independent. In the present study, centralities of catatonia signs and global functioning emerged numerically, but not significantly smaller than NSS centrality, suggesting similarly strong connections of NSS, catatonia signs as well as global functioning with all other network variables. On the one hand, for catatonia signs, this connectivity was mainly driven by associations with global functioning and parkinsonism symptoms. On the other hand, for global functioning, this connectivity was mainly driven by associations with psychopathology and catatonia signs. Overall, the above mentioned centralities emphasize the intertwined nature of NSS, catatonia signs, and global functioning.

Third, our result showing close connections of NSS and cognition is consistent with other studies^78–80^. However, the techniques employed in these prior studies were constrained since they did not have the capability to consider the dynamic impact of other pertinent factors on the focal connection of NSS and cognition. In addition, there have been reports of connections between different sensorimotor abnormalities and psychopathological domains^78^. Yet, our network approach incorporates the relationships between the sensori- and psychomotor abnormalities and cognitive domains, while simultaneously taking into consideration psychopathological symptoms, global functioning, and antipsychotic medication dose. Furthermore, the present findings indicate that the correlation between NSS and cognition was significantly stronger than the correlation between other sensori- and psychomotor abnormalities and cognition. Here, we hypothesize a shared underlying pathology between NSS and cognition, which requires investigation in future studies^17,81,82^ and could lead to the identification of novel treatment targets for cognitive deficits in SSD. For example, the Heidelberg NSS contains the items Ozeretzki´s test and fist-edge-palm test, which require explanation and practice before rating. Participants with lower cognitive functioning may perform worse in these tasks not only because of lower sensorimotor, but also because of lower cognitive functioning. Of interest, as shown by separate Spearman correlations in this dataset (see Supplementary Table 2), NSS examination involved more cognitive effort than other sensori- and psychomotor abnormalities.

Fourth, catatonia signs were strongly correlated with global functioning. In line with this, patients with catatonia frequently display significantly diminished GAF scores^83,84^, perhaps as a result of the severe influence of catatonia symptoms on their daily functioning and general quality of life. In comparison to patients without catatonic features, individuals with catatonia and mania had more severe mixed and manic symptoms, higher levels of general psychopathology and comorbidity, longer hospital stays and worse GAF scores^83^. Furthermore, when comparing individuals with and without catatonia, lower scores on the GAF and SOFAS measures were shown^84^. Moreover, in a retrospective analysis of SSD patients, catatonia was correlated with a 7.4 point decrease in global functioning^85^. More recently, it was shown that the Northoff Scale for Subjective Experience in Catatonia (NSSC) scores were significantly associated with lower GAF scores in catatonia patients according to ICD-11^45^. This finding emphasizes that it is probably not only the externally visible catatonic signs, but also the subjective experience of these conditions that can lead to a reduced level of global functioning. The present result associating catatonia signs with lower GAF scores extends these findings by simultaneously controlling for the effects of sensori- and psychomotor abnormalities as well as for psychopathology, cognition and daily antipsychotic medication dose. While it was hitherto unclear, for example, whether the association between catatonia signs and lower GAF scores may have been mediated by, e.g., psychopathology, cognition or daily antipsychotic medication dose, our result suggests that this may not be the case.

This independence of antidopaminergic treatment effects is also confirmed by the network analysis showing that daily antipsychotic medication had low centrality and weak connections with all sensori- and psychomotor abnormalities. This finding also reinforces the notion that these symptom clusters are part of the core pathophysiology of SSD.

Interestingly, in the analysis focusing solely on sensori- and psychomotor nodes, key network characteristics aligned with those observed in the broader analyses including PANSS total score, OLZe, GAF, and B-CATS. Notably, the strong connection between NSS and SAS remained prominent, and the connection between AIMS and BARS was consistently stronger than their connections with other nodes. Regarding centrality, expected influence emerged as the only stable centrality parameter across conditions, highlighting the robustness of these findings irrespective of adjustments for age, sex, and education.

Of note, based on nodewise predictability, OLZe displayed a nodewise predictability of *R*² = 0, indicating that it is not determined by other variables in the network. This finding raises an intriguing question about the extent to which OLZe influences other nodes. However, as far as we are aware, nodewise predictability provides information only on how much a node is determined by other nodes, not on how much a node affects other nodes. This distinction is relevant given the traditional view that antipsychotic medication is a primary cause of sensori- and psychomotor abnormalities. More recent findings, however, suggest that these abnormalities are intrinsic to the pathophysiology of SSD. Further, the low to moderate nodewise predictability observed in our network suggest that, despite overlapping signs and symptoms, the five sensori- and psychomotor abnormalities included in this study are distinct from one another to varying degrees. This supports the notion of their complex and multifaceted interrelations, emphasizing the need for further research into their individual and shared contributions to SSD pathophysiology.

### Strengths and limitations

The first application of network analysis techniques to the research question of the relationship between sensori- and psychomotor abnormalities and cognition and functioning, the study sample size and a solid theoretical framework as well as controlling for other variables related to cognition and functioning in this network analytic framework represent main strengths of this study.

However, several limitations should also be taken into account: First, throughout this manuscript we employed the terms sensori- (NSS, parkinsonism, dyskinesia, and akathisia) and psychomotor (catatonia) abnormalities. However, the boundaries between sensori- and psychomotor abnormalities are arbitrary, largely influenced by the use of different clinical rating scales and subject of further studies. Further, we acknowledge the complexity and overlap between sensori- and psychomotor abnormalities in SSD, both clinically (rigidity, restlessness, stereotypy, etc.) and neurobiologically. Shared brain circuits may contribute to the observed intersections between these phenomena, making a clear boundary challenging to define. For example, akathisia often combines subjective affective experiences with observable motor restlessness and aggression, while catatonia encompasses motor, affective, and behavioral components. Despite these overlaps, we argue that distinguishing between sensori- and psychomotor abnormalities remains clinically and neurobiologically plausible. Catatonia, for instance, is widely recognized as a psychomotor disorder with its own distinct criteria, a perspective supported by historical literature and codified in the ICD-11 classification. While it might be simpler to group all sensori- and psychomotor abnormalities under the umbrella of “motor disorders,” as suggested by Daniel Roger’s “conflict of paradigms”^86^, doing so risks oversimplifying the clinical picture. We acknowledge the limitations of the current phenomenological framework regarding the symptom/sign overlap and the need for further refinement. Still, we believe that a more nuanced categorization offers crucial insights into underlying mechanisms and supports the development of more targeted treatment approaches.

A second potential limitation of our study lies in its cross-sectional design, which does not allow for modeling changes over time or testing causal relationships. While cross-sectional analyses can generate valuable hypotheses, they are inherently limited in their ability to confirm causality, which requires longitudinal study designs. Additionally, our focus on calculating linear relationships within the network may overlook complex, non-linear associations that could more accurately describe the interactions between variables. Although alternative centrality metrics such as betweenness and closeness centrality could offer insights into different network properties, their stability coefficients were not reliable in our analysis and, therefore, could not be interpreted. This restricted our analysis to strength and expected influence (EI) centrality. Furthermore, network structures estimated across a study sample may not fully represent individual-level networks, particularly given the potential variability in network configurations among patients with SSD.

Third, we included two cohorts that were assessed during different time periods and by different examiners. The identified differences between Cohort #1 and Cohort #2 (see Supplementary Table 1) regarding sex, OLZe, GAF, NCRS, SAS, BARS, and the Digit Symbol Substitution Test (a subtest of the B-CATS) could introduce variability that may influence the results. However, given that large consortia like the Human Connectome Project or UK Biobank do not provide detailed assessments of sensori- and psychomotor abnormalities, we believe that the two cohorts complement each other by capturing distinct but overlapping facets of these abnormalities. Together, they offer a naturalistic and real-world representation of SSD patients in Germany. Combining these cohorts was also essential to enhance the study’s statistical power while preserving its clinical relevance. In this context, performing sensitivity analyses, i.e., repeating the analyses in each cohort separately, was not possible due to the low number of participants in the individual cohorts in terms of network analysis.

Fourth, a potential limitation of our study is the inability to conduct network analyses at the item- or subscale-level due to sample size constraints. While previous network analyses^87,88^ have demonstrated that examining data on multiple levels, including subscores and single-item relationships, can yield more detailed insights, our hypotheses focused on broader clusters of sensori- and psychomotor abnormalities, represented by total scores. Including a larger number of variables, such as individual symptom/signs items across different rating scales, would have increased the complexity of network estimation, requiring a substantially larger sample size to ensure stability of a network with more than 25 nodes. Consequently, we limited our analysis to broader domains, including sensorimotor, psychomotor, psychopathological, cognitive, and functional aspects, rather than granular item-level interactions. We strongly recommend that future studies with larger cohorts explore more detailed networks to further enhance our understanding of these relationships.

Fifth, the low average values of TD and akathisia (and their low prevalence) may contribute to their weak connections and low centrality in the network. In particular, the majority of participants did not fulfill the criteria for TD or akathisia. In that regard, complementary studies should be conducted, focusing on participants fulfilling categorically defined sensori- and psychomotor abnormalities. In this network analysis, we focused on a dimensional approach including the full severity spectrum of sensori- and psychomotor abnormalities in SSD. Sixth, the use of olanzapine equivalents^63^ to standardize antipsychotic dosages may have introduced a potential confounding factor in the results. While this method is a well-established approach to facilitate comparisons across different medications, it may not fully capture the unique effects of individual antipsychotics on sensori- and psychomotor functions. Given the differential risk profiles of various antipsychotics—such as the higher likelihood of motor side effects with first-generation agents and risperidone compared to the lower risk with clozapine and quetiapine—our network analysis might be influenced by this standardization. Further, the German healthcare system does not support a centralized patient registry. This, combined with the challenges many patients face in accurately recalling their medication history, meant that we could only assess the influence of current daily antipsychotic dose. While the vast majority of patients were treated according to the German S3 guideline with second-generation antipsychotics, this limitation may have influenced our findings. Nevertheless, prior studies, including the longitudinal work by Parksepp et al. ^89^, have shown that antipsychotic dose does not significantly affect the severity of antipsychotic-induced motor abnormalities. Additionally, a recent meta-analysis from the Cochrane Database of Systematic Reviews reported that the prevalence of movement abnormalities associated with antipsychotics was not influenced by treatment duration^90^. These findings support the interpretation that medication dose may play a less significant role in sensori- and psychomotor abnormalities than previously assumed. Finally, the naturalistic design of our study aimed to enhance its generalizability and real-world relevance, although this approach introduces variability that may limit the ability to control for all potential confounding factors. Future research should consider utilizing longitudinal designs with more comprehensive medication tracking to address these limitations. In addition, patients were recruited after achieving partial remission of acute symptoms based on clinical assessment, with the duration of remission varying. In addition, the ongoing adjustment of antipsychotic medication, particularly in inpatient settings, could be a potential confounding factor that could influence the results.

Finally, nearly all SSD patients exhibited some form of NSS, which could have led to an overestimation of the centrality of NSS in the network, necessitating further replication to validate these findings. This overrepresentation may not be generalizable to other samples with lower NSS prevalence. For comparison, Bachmann et al.^47^ studied 39 first-episode SSD patients (mean age 27.0 years, all in remission) and reported a mean total Heidelberg NSS Scale Score of 15.7. In contrast, the present study’s higher mean total score of 20.4 on the Heidelberg NSS Scale might largely be attributed to the characteristics of our sample (mean age 37.75, participants in partial remission). This interpretation is supported by Manschreck et al.^91^, who documented a 92% prevalence of NSS in schizophrenia, and the observation that NSS may increase with age^92^. While such an increase of NSS prevalence or severity with age could be a treatment effect, our results seem to suggest that NSS are both a trait but also state phenomenon that may worsen with advancing schizophrenia illness and its underlying neurobiological basis. Furthermore, we have refrained from carrying out a sensitivity analysis, because setting an arbitrary cut-off score to form a validation cohort would have been artificial and scientifically unsound, as there are no established thresholds for NSS rating scales in the literature. The lack of a clear cut-off score complicates interpretation and could lead to bias, as such an arbitrary threshold would not accurately reflect the continuous and neurodevelopmental nature of NSS. Therefore, without a solid cut-off value supported by the literature, any sensitivity analysis would not provide valid or reliable findings. Nevertheless, despite these limitations, this study is to our knowledge the first network analysis that investigated the relationship between sensori- and psychomotor abnormalities and cognition and functioning controlling for other relevant variables.

### Closing the research gaps: recommendation for future studies

The findings of this study underscore the profound interdependence and clinical importance of sensori- and psychomotor abnormalities in SSD. At the same time, they highlight significant gaps in our current understanding, offering valuable opportunities for future research. These sensori- and psychomotor abnormalities represent a complex spectrum of dysfunctions that are deeply intertwined yet not fully delineated. Here we discuss some important aspects of research on this clinically relevant domain:

1. Rethinking Constructs and Boundaries: The boundaries between sensori- and psychomotor abnormalities remain largely arbitrary, shaped by the use of distinct clinical rating scales that often capture overlapping signs and symptoms. To better understand the boundaries between the sensori- and psychomotor systems, we recommend that future studies aim to rethink and refine these constructs. One approach could involve conducting longitudinal large-scale cohort studies that utilize comprehensive assessments from all available rating scales —including NSS, parkinsonism, akathisia, dyskinesia, and catatonia— and complement them with motion-capturing technology^93^ to capture the full spectrum of sensori- and psychomotor abnormalities. By employing unsupervised machine learning techniques such as hierarchical clustering or network analysis techniques on total scores, subscores and single-item level, researchers can explore the underlying structure of sensori- and psychomotor signs and symptoms. Specifically, the former method may yield distinct symptom/sign clusters, while the latter approach using community detection methods such as exploratory graph analysis within the network analysis framework could help to identify whether sensori- and psychomotor abnormalities form distinct communities. Hypothetically, these approaches may reveal two separate groups: one reflecting sensorimotor abnormalities and another reflecting psychomotor abnormalities. Importantly, integrating brain imaging data into these analyses could further elucidate the neural networks associated with each group, potentially linking them to specific sensori- and psychomotor-associated brain networks. Another approach could focus on identifying the neural networks underlying categorically-defined sensorimotor and psychomotor abnormalities, preferably combining different MRI modalities such as diffusion tensor imaging and functional MRI. Finally, we acknowledge that our analyses were not sufficiently powered to perform subscale analyses or analyses at the item level. Therefore, we recommend that future multi-site longitudinal studies should include detailed sensori- and psychomotor assessments also in order to take into account the established factor structure of the PANSS^94,95^.
2. Neural Correlates and Mechanistic Understanding: Integrating neuroimaging data into these network analyses could further enhance our understanding by linking clinical symptoms/signs to specific neural networks. Identifying the brain regions and circuits associated with sensori- and psychomotor abnormalities could pave the way for a mechanistic understanding of these symptoms. For instance, distinct brain networks associated with sensori- and psychomotor functions could guide the development of more targeted therapeutic strategies. Our previous work on NSS subscales in larger samples, such as the studies by Fritze et al.^1,33^, exemplified how detailed analyses within subsets of abnormalities can yield valuable insights when adequate sample sizes are available.
3. Implications for Diagnosis and Treatment: The observed overlap between sensori- and psychomotor abnormalities also raises important questions about their implications for diagnostic and therapeutic strategies in SSD. The strong centrality of nodes such as NSS, catatonia signs, and global functioning in our study suggests that these features could serve as pivotal treatment targets. Interventions addressing central nodes within a network could potentially improve the entire system, aligning with the principles of precision medicine. Future research should explore the integration of neurobiologically plausible brain regions as network nodes, linking clinical symptoms/signs to underlying neural mechanisms. Such an approach could inform the development of targeted interventions, including pharmacological treatments and non-invasive brain stimulation techniques like transcranial magnetic stimulation (TMS) or transcranial direct current stimulation (tDCS).
4. Longitudinal Dynamics and Personalized Interventions: Longitudinal studies are crucial for understanding the dynamics of these networks over time. By conducting network analyses at multiple time points, such as at the initiation and end of therapy, researchers can evaluate how treatments influence the structure and centrality of sensori- and psychomotor nodes. This could help to identify the most effective interventions and refine personalized treatment strategies based on network changes induced by specific therapies. Such designs would also provide insights into the progression of sensori- and psychomotor abnormalities and their response to therapeutic interventions.
5. Subgroup-specific research and future clinical trials: The independence of sensori- and psychomotor abnormalities from antipsychotic medication, as observed in our findings, is an intriguing result that warrants further investigation. Future studies should explore whether specific subgroups of patients might benefit from pharmacological or non-pharmacological interventions targeting these abnormalities. Such studies would require robust longitudinal or interventional designs to account for treatment effects and patient variability. Furthermore, clinical trials should consider including sensori- and psychomotor network nodes as primary or secondary endpoints to systematically evaluate the effects of treatments on network structure and function.
6. Toward a Mechanism-Based Approach: By linking these network nodes to targeted brain regions, researchers could develop novel network-guided therapies that combine pharmacological approaches with non-invasive brain stimulation techniques (rTMS and tDCS), optimizing treatment outcomes. The integration of longitudinal designs, network-based analyses, and targeted interventions will be critical to advancing our understanding of sensori- and psychomotor abnormalities in SSD. These efforts could significantly enhance diagnostic precision and therapeutic efficacy, driving the field toward a more personalized and mechanism-based approach to care.

## Conclusion

This study, to our knowledge, for the first time modeled and quantified distinct interaction patterns between sensori- and psychomotor abnormalities and their relationship to cognition and functioning in SSD. Preferential connections occurred between NSS and cognition, parkinsonism and NSS as well as between catatonia signs and global functioning. It will be fruitful that forthcoming studies incorporate these nuanced interrelationships to enhance the understanding and treatment of these conditions.

## Supplementary information

Supplementary material

## Data Availability

The data of this study are available from the corresponding author upon reasonable request.

## References

[1] Fritze, S. et al. Characterizing the sensorimotor domain in schizophrenia spectrum disorders. *Eur. Arch. Psychiatry Clin. Neurosci.* **272**, 1097–1108 (2022). 34839404 10.1007/s00406-021-01354-9 PMC9388408

[2] Hirjak, D., Meyer-Lindenberg, A., Sambataro, F. & Christian Wolf, R. Sensorimotor neuroscience in mental disorders: progress, perspectives and challenges. *Schizophr. Bull.* **47**, 880–882 (2021). 33940630 10.1093/schbul/sbab053 PMC8266597

[3] Hirjak, D. et al. Progress in sensorimotor neuroscience of schizophrenia spectrum disorders: Lessons learned and future directions. *Prog. Neuro-Psychopharmacol. Biol. Psychiatry* **111**, 110370 (2021). 10.1016/j.pnpbp.2021.110370 34087392

[4] Siafis, S., Wu, H., Wang, D. & Burschinski, A. Antipsychotic dose, dopamine D2 receptor occupancy and extrapyramidal side-effects: a systematic review and dose-response meta-analysis. *Mol. Psychiatry* **28**, 3267–3277 (2023). 37537284 10.1038/s41380-023-02203-y PMC10618092

[5] Solmi, M., Pigato, G., Kane, J. M. & Correll, C. U. Clinical risk factors for the development of tardive dyskinesia. *J. Neurol. Sci.* **389**, 21–27 (2018). 29439776 10.1016/j.jns.2018.02.012

[6] Wu, H. et al. Antipsychotic-induced akathisia in adults with acute schizophrenia: a systematic review and dose-response meta-analysis. *Eur. Neuropsychopharmacol.* **72**, 40–49 (2023). 37075639 10.1016/j.euroneuro.2023.03.015

[7] Solmi, M. et al. Prevalence of catatonia and its moderators in clinical samples: results from a meta-analysis and meta-regression analysis. *Schizophr. Bull.* **44**, 1133–1150 (2018). 29140521 10.1093/schbul/sbx157 PMC6101628

[8] Bernard, J. A. & Mittal, V. A. Updating the research domain criteria: the utility of a motor dimension. *Psychol. Med.* **45**, 2685–2689 (2015). 26005109 10.1017/S0033291715000872 PMC4565742

[9] Mittal, V. A., Bernard, J. A. & Northoff, G. What can different motor circuits tell us about psychosis? An RDoC perspective. *Schizophr. Bull.* **43**, 949–955 (2017). 28911048 10.1093/schbul/sbx087 PMC5581904

[10] Northoff, G., Hirjak, D., Wolf, R. C., Magioncalda, P. & Martino, M. Why is there symptom coupling of psychological and motor changes in psychomotor mechanisms? Insights from the brain’s topography. *Mol. Psychiatry* **26**, 3669–3671 (2021). 33203994 10.1038/s41380-020-00945-7

[11] Northoff, G., Hirjak, D., Wolf, R. C., Magioncalda, P. & Martino, M. All roads lead to the motor cortex: psychomotor mechanisms and their biochemical modulation in psychiatric disorders. *Mol. Psychiatry* **26**, 92–102 (2021). 32555423 10.1038/s41380-020-0814-5

[12] Hirjak, D. et al. Patterns of co-altered brain structure and function underlying neurological soft signs in schizophrenia spectrum disorders. *Hum. Brain Mapp.* **40**, 5029–5041 (2019). 31403239 10.1002/hbm.24755 PMC6865492

[13] Hirjak, D. et al. White matter microstructure variations contribute to neurological soft signs in healthy adults. *Hum. Brain Mapp.* **38**, 3552–3565 (2017). 28429448 10.1002/hbm.23609 PMC6867126

[14] Hirjak, D., Wolf, R. C., Kubera, K. M., Stieltjes, B. & Thomann, P. A. Multiparametric mapping of neurological soft signs in healthy adults. *Brain Struct. Funct.* **221**, 1209–1221 (2016). 25528225 10.1007/s00429-014-0964-9

[15] Wolf, R. C. et al. Neurological soft signs predict auditory verbal hallucinations in patients with schizophrenia. *Schizophr. Bull.* **47**, 433–443 (2021). 33097950 10.1093/schbul/sbaa146 PMC7965075

[16] Emsley, R. et al. Neurological soft signs in first-episode schizophrenia: state- and trait-related relationships to psychopathology, cognition and antipsychotic medication effects. *Schizophr. Res.* **188**, 144–150 (2017). 28130002 10.1016/j.schres.2017.01.034

[17] Zhao, Q. et al. Neurological soft signs are not “soft” in brain structure and functional networks: evidence from ALE meta-analysis. *Schizophr. Bull.* **40**, 626–641 (2014). 23671197 10.1093/schbul/sbt063 PMC3984512

[18] Carbon, M., Kane, J. M., Leucht, S. & Correll, C. U. Tardive dyskinesia risk with first- and second-generation antipsychotics in comparative randomized controlled trials: a meta-analysis. *World Psychiatry* **17**, 330–340 (2018). 30192088 10.1002/wps.20579 PMC6127753

[19] Correll, C. U., Kane, J. M. & Citrome, L. L. Epidemiology, prevention, and assessment of tardive dyskinesia and advances in treatment. *J. Clin. Psychiatry* **78**, 1136–1147 (2017). 29022654 10.4088/JCP.tv17016ah4c

[20] Carbon, M., Hsieh, C. H., Kane, J. M. & Correll, C. U. Tardive dyskinesia prevalence in the period of second-generation antipsychotic use: a meta-analysis. *J. Clin. Psychiatry* **78**, e264–e278 (2017). 28146614 10.4088/JCP.16r10832

[21] Cuesta, M. J. et al. Spontaneous parkinsonism is associated with cognitive impairment in antipsychotic-naive patients with first-episode psychosis: a 6-month follow-up study. *Schizophr. Bull.* **40**, 1164–1173 (2014). 24072809 10.1093/schbul/sbt125 PMC4133659

[22] Schoretsanitis, G., Nikolakopoulou, A., Guinart, D., Correll, C. U. & Kane, J. M. Iron homeostasis alterations and risk for akathisia in patients treated with antipsychotics: a systematic review and meta-analysis of cross-sectional studies. *Eur. Neuropsychopharmacol.* **35**, 1–11 (2020). 32444336 10.1016/j.euroneuro.2020.04.001

[23] Kane, J. M. et al. Evaluation of akathisia in patients with schizophrenia, schizoaffective disorder, or bipolar I disorder: a post hoc analysis of pooled data from short- and long-term aripiprazole trials. *J. Psychopharmacol.* **24**, 1019–1029 (2010). 20008446 10.1177/0269881109348157

[24] Fritze, S. et al. Neurological soft signs in schizophrenia spectrum disorders are not confounded by current antipsychotic dosage. *Eur. Neuropsychopharmacol.* **31**, 47–57 (2020). 31780303 10.1016/j.euroneuro.2019.11.001

[25] Heuser, M., Thomann, P. A., Essig, M., Bachmann, S. & Schroder, J. Neurological signs and morphological cerebral changes in schizophrenia: an analysis of NSS subscales in patients with first-episode psychosis. *Psychiatry Res.* **192**, 69–76 (2011). 21498055 10.1016/j.pscychresns.2010.11.009

[26] Mouchet-Mages, S. et al. Correlations of cerebello-thalamo-prefrontal structure and neurological soft signs in patients with first-episode psychosis. *Acta Psychiatr. Scand.* **123**, 451–458 (2011). 21219267 10.1111/j.1600-0447.2010.01667.x

[27] Hirjak, D. et al. Neurological soft signs and brainstem morphology in first-episode schizophrenia. *Neuropsychobiology* **68**, 91–99 (2013). 23881157 10.1159/000350999

[28] Rossi A., *et al*. Neurological soft signs in schizophrenia. *Br. J. Psychiatry* **157**, 735–739 (1990). 10.1192/bjp.157.5.735 2132562

[29] Lane, A. et al. Schizophrenia and neurological soft signs: gender differences in clinical correlates and antecedent factors. *Psychiatry Res.* **64**, 105–114 (1996). 8912952 10.1016/0165-1781(96)02602-9

[30] Kong, L., Bachmann, S., Thomann, P. A., Essig, M. & Schroder, J. Neurological soft signs and gray matter changes: a longitudinal analysis in first-episode schizophrenia. *Schizophr. Res.* **134**, 27–32 (2012). 22018942 10.1016/j.schres.2011.09.015

[31] Cuesta, M. J. et al. Motor abnormalities in first-episode psychosis patients and long-term psychosocial functioning. *Schizophr. Res.* **200**, 97–103 (2018). 28890132 10.1016/j.schres.2017.08.050

[32] Pieters, L. E., Nadesalingam, N., Walther, S. & van Harten, P. N. A systematic review of the prognostic value of motor abnormalities on clinical outcome in psychosis. *Neurosci. Biobehav. Rev.* **132**, 691–705 (2022). 34813828 10.1016/j.neubiorev.2021.11.027

[33] Fritze, S. et al. Deciphering the interplay between psychopathological symptoms, sensorimotor, cognitive and global functioning: a transdiagnostic network analysis. *Eur. Arch. Psychiatry Clin. Neurosci.* **274**, 1625–1637 (2024). 38509230 10.1007/s00406-024-01782-3 PMC11422259

[34] Walther, S. & Mittal, V. A. Why we should take a closer look at gestures. *Schizophr. Bull.* **42**, 259–261 (2016). 26773476 10.1093/schbul/sbv229 PMC4753618

[35] Peralta, V., de Jalón, E. G., Campos, M. S. & Cuesta, M. J. Covariation between motor signs and negative symptoms in drug-naive subjects with schizophrenia-spectrum disorders before and after antipsychotic treatment. *Schizophr. Res.* **200**, 85–91 (2018). 28864283 10.1016/j.schres.2017.08.039

[36] Walther, S. & Strik, W. Motor symptoms and schizophrenia. *Neuropsychobiology* **66**, 77–92 (2012). 22814247 10.1159/000339456

[37] Borsboom, D. A network theory of mental disorders. *World Psychiatry* **16**, 5–13 (2017). 28127906 10.1002/wps.20375 PMC5269502

[38] Borsboom, D. et al. Network analysis of multivariate data in psychological science. *Nat. Rev. Methods Prim.* **1**, 58 (2021).

[39] Sass H., Wittchen H. U., Zaudig M., I. H. *Diagnostisches und Statistisches Manual Psychischer Störungen DSM-IV-TR: Textrevision*. 1001 (Hogrefe Verlag; 2003).

[40] Northoff, G. et al. Cortical morphology and illness insight in patients with schizophrenia. *Hum. Brain Mapp.* **272**, 985–995 (2022). 10.1007/s00406-021-01328-x PMC9388450 34518921

[41] Sambataro, F. & Hirjak, D. Intrinsic neural network dynamics in catatonia. *Hum. Brain Mapp.* **42**, 6087–6098 (2021). 34585808 10.1002/hbm.25671 PMC8596986

[42] Hirjak, D. et al. Microstructural white matter biomarkers of symptom severity and therapy outcome in catatonia: Rationale, study design and preliminary clinical data of the whiteCAT study. *Schizophr. Res* **263**, 160–168 (2024). 37236889 10.1016/j.schres.2023.05.011

[43] Margraf J., Schneider S., Ehlers A., Psychologie C-D-SfK. *Diagnostisches Interview bei Psychischen Störungen: DIPS* (Springer, 1994).

[44] Peretzke, R. et al. Deciphering white matter microstructural alterations in catatonia according to ICD-11: replication and machine learning analysis. Mol Psychiatry. 10.1038/s41380-024-02821-0. 10.1038/s41380-024-02821-0 PMC12014485 39623072

[45] Brandt, GA. et al. Extension, translation and preliminary validation of the Northoff Scale for Subjective Experience in Catatonia (NSSC). *Schizophr Res.* **263**, 282–288 (2024). 37331880 10.1016/j.schres.2023.06.002

[46] Schröder, J. et al. Neurological soft signs in schizophrenia. *Schizophr. Res.* **6**, 25–30 (1991). 1786233 10.1016/0920-9964(91)90017-l

[47] Bachmann, S., Bottmer, C. & Schröder, J. Neurological soft signs in first-episode schizophrenia: a follow-up study. *Am. J. Psychiatry* **162**, 2337–2343 (2005). 16330599 10.1176/appi.ajp.162.12.2337

[48] Simpson, G. M. & Angus, J. W. A rating scale for extrapyramidal side effects. *Acta Psychiatr. Scand. Suppl.* **212**, 11–19 (1970). 4917967 10.1111/j.1600-0447.1970.tb02066.x

[49] Hirjak D., Thomann P. A., Northoff G., Kubera K. M., Wolf R. C. NCR-Skala—Deutsche Version der Northoff Catatonia Rating Scale (NCRS-dv)—Ein validiertes Messinstrument zur Erfassung katatoner Symptome. *Der Nervenarzt* (im Druck) (2016). 10.1007/s00115-016-0136-7 27325247

[50] Barnes, T. R. The Barnes Akathisia Rating Scale-revisited. *J. Psychopharmacol.* **17**, 365–370 (2003). 14870947 10.1177/0269881103174013

[51] Guy W. Abnormal involuntary movement scale (AIMS). *ECDEU Assessment Manual for Psychopharmacology* (1976).

[52] Thomann, P. A., Hirjak, D., Kubera, K. M., Stieltjes, B. & Wolf, R. C. Neural network activity and neurological soft signs in healthy adults. *Behav. Brain Res.* **278**, 514–519 (2015). 25446752 10.1016/j.bbr.2014.10.044

[53] Peralta, V., de Jalón, E. G., Campos, M. S. & Cuesta, M. J. Phenomenological differences between spontaneous and drug-related extrapyramidal syndromes in patients with schizophrenia-spectrum disorders. *J. Clin. Psychopharmacol.* **33**, 438–440 (2013). 23609391 10.1097/JCP.0b013e31828f62b0

[54] Hirjak, D. et al. Multimodal magnetic resonance imaging data fusion reveals distinct patterns of abnormal brain structure and function in catatonia. *Schizophr. Bull.* **46**, 202–210 (2020). 31174212 10.1093/schbul/sbz042 PMC6942158

[55] Wasserthal, J. et al. Multiparametric mapping of white matter microstructure in catatonia. *Neuropsychopharmacology* **45**, 1750–1757 (2020). 32369829 10.1038/s41386-020-0691-2 PMC7419514

[56] Janno, S., Holi, M., Tuisku, K. & Wahlbeck, K. Prevalence of neuroleptic-induced movement disorders in chronic schizophrenia inpatients. *Am. J. Psychiatry* **161**, 160–163 (2004). 14702266 10.1176/appi.ajp.161.1.160

[57] Gopal, S. et al. Incidence of tardive dyskinesia: a comparison of long-acting injectable and oral paliperidone clinical trial databases. *Int. J. Clin. Pract.* **68**, 1514–1522 (2014). 25358867 10.1111/ijcp.12493 PMC4265240

[58] Schooler, N. R. & Kane, J. M. Research diagnoses for tardive dyskinesia. *Arch. Gen. Psychiatry* **39**, 486–487 (1982). 6121550 10.1001/archpsyc.1982.04290040080014

[59] Kay, S. R., Fiszbein, A. & Opler, L. A. The positive and negative syndrome scale (PANSS) for schizophrenia. *Schizophr. Bull.* **13**, 261–276 (1987). 3616518 10.1093/schbul/13.2.261

[60] Hurford, I. M., Marder, S. R., Keefe, R. S., Reise, S. P. & Bilder, R. M. A brief cognitive assessment tool for schizophrenia: construction of a tool for clinicians. *Schizophr. Bull.* **37**, 538–545 (2011). 19776205 10.1093/schbul/sbp095 PMC3080688

[61] DSM-III.R. DKuDddusMpSr Gaf-skala: Global Assessment of Functioning Scale (Beltz, 1989).

[62] R Core Team. R: A Language and Environment for Statistical Computing. https://www.R-project.org/ (R Foundation for Statistical Computing, 2021).

[63] Leucht, S. et al. Dose equivalents for second-generation antipsychotic drugs: the classical mean dose method. *Schizophr. Bull.* **41**, 1397–1402 (2015). 25841041 10.1093/schbul/sbv037 PMC4601707

[64] Costantini, G. et al. State of the aRt personality research: a tutorial on network analysis of personality data in R. *J. Res. Personal.* **54**, 13–29 (2015).

[65] Hevey, D. Network analysis: a brief overview and tutorial. *Health Psychol. Behav. Med.* **6**, 301–328 (2018). 34040834 10.1080/21642850.2018.1521283 PMC8114409

[66] Epskamp, S., Borsboom, D. & Fried, E. I. Estimating psychological networks and their accuracy: a tutorial paper. *Behav. Res. Methods* **50**, 195–212 (2018). 28342071 10.3758/s13428-017-0862-1 PMC5809547

[67] Epskamp, S. & Fried, E. I. A tutorial on regularized partial correlation networks. *Psychol. Methods* **23**, 617–634 (2018). 29595293 10.1037/met0000167

[68] Epskamp, S., Cramer, A. O. J., Waldorp, L. J., Schmittmann, V. D. & Borsboom, D. qgraph: network visualizations of relationships in psychometric data. *J. Stat. Softw.* **48**, 1–18 (2012).

[69] Haslbeck, J. M. & Waldorp, L. J. How well do network models predict observations? On the importance of predictability in network models. *Behav. Res. Methods* **50**, 853–861 (2018). 28718088 10.3758/s13428-017-0910-x PMC5880858

[70] Chen, K. W., Killeya-Jones, L. A. & Vega, W. A. Prevalence and co-occurrence of psychiatric symptom clusters in the U.S. adolescent population using DISC predictive scales. *Clin. Pract. Epidemiol. Ment. Health* **1**, 22 (2005). 16255774 10.1186/1745-0179-1-22 PMC1298317

[71] Lallukka, T. et al. Co-occurrence of depressive, anxiety, and somatic symptoms: trajectories from adolescence to midlife using group-based joint trajectory analysis. *BMC Psychiatry* **19**, 236 (2019). 31370894 10.1186/s12888-019-2203-7 PMC6670180

[72] Peralta, V. & Cuesta, M. J. Neuromotor abnormalities in neuroleptic-naive psychotic patients: antecedents, clinical correlates, and prediction of treatment response. *Compr. Psychiatry* **52**, 139–145 (2011). 21295219 10.1016/j.comppsych.2010.05.009

[73] Hirjak, D., Meyer-Lindenberg, A., Kubera, KM., Thomann, PA. & Wolf, RC. Motor dysfunction as research domain in the period preceding manifest schizophrenia: A systematic review. *Neurosci Biobehav Rev.* **87**, 87–105 (2018). 29410313 10.1016/j.neubiorev.2018.01.011

[74] Hirjak, D. et al. Neuroanatomical markers of neurological soft signs in recent-onset schizophrenia and asperger-syndrome. *Brain Topogr.* **29**, 382–394 (2016). 26708327 10.1007/s10548-015-0468-9

[75] Wasserthal, J. et al. White matter microstructure alterations in cortico-striatal networks are associated with parkinsonism in schizophrenia spectrum disorders. *Eur. Neuropsychopharmacol.* **50**, 64–74 (2021). 33984810 10.1016/j.euroneuro.2021.04.007

[76] Wolf, R. C. et al. A neural signature of parkinsonism in patients with schizophrenia spectrum disorders: a multimodal MRI study using parallel ICA. *Schizophr. Bull.* **46**, 999–1008 (2020). 32162660 10.1093/schbul/sbaa007 PMC7345812

[77] Bernard, J. A. & Mittal, V. A. Cerebellar-motor dysfunction in schizophrenia and psychosis-risk: the importance of regional cerebellar analysis approaches. *Front. Psychiatry* **5**, 160 (2014). 25505424 10.3389/fpsyt.2014.00160 PMC4243486

[78] Sambataro F., *et al*. Moving forward: distinct sensorimotor abnormalities predict clinical outcome after 6 months in patients with schizophrenia. *Eur. Neuropsychopharmacol.* **36**, 72–82 (2020). 10.1016/j.euroneuro.2020.05.002 32522386

[79] Herold, C. J., Duval, C. Z. & Schröder, J. Neurological soft signs and cognition in the late course of chronic schizophrenia: a longitudinal study. *Eur. Arch. Psychiatry Clin. Neurosci.* **271**, 1465–1473 (2021). 32417958 10.1007/s00406-020-01138-7 PMC8563630

[80] Bachmann, S. & Schröder, J. Neurological soft signs in schizophrenia: an update on the state- versus trait-perspective. *Front. Psychiatry* **8**, 272 (2017). 29375401 10.3389/fpsyt.2017.00272 PMC5766896

[81] Gay, O. et al. Cognitive control deficit in patients with first-episode schizophrenia is associated with complex deviations of early brain development. *J. Psychiatry Neurosci.* **42**, 87–94 (2017). 28245174 10.1503/jpn.150267 PMC5373705

[82] Rusch, N. et al. Prefrontal-thalamic-cerebellar gray matter networks and executive functioning in schizophrenia. *Schizophr. Res.* **93**, 79–89 (2007). 17383859 10.1016/j.schres.2007.01.029

[83] Braunig, P., Kruger, S. & Shugar, G. Prevalence and clinical significance of catatonic symptoms in mania. *Compr. Psychiatry* **39**, 35–46 (1998). 9472454 10.1016/s0010-440x(98)90030-x

[84] Nadesalingam, N. et al. Motor abnormalities are associated with poor social and functional outcomes in schizophrenia. *Compr. Psychiatry* **115**, 152307 (2022). 35303585 10.1016/j.comppsych.2022.152307

[85] Kline, C. L., Suzuki, T., Simmonite, M. & Taylor, S. F. Catatonia is associated with higher rates of negative affect amongst patients with schizophrenia and schizoaffective disorder. *Schizophr. Res.* **263**, 208–213 (2024). 36114099 10.1016/j.schres.2022.09.001

[86] Rogers, D. The motor disorders of severe psychiatric illness: a conflict of paradigms. *Br. J. Psychiatry* **147**, 221–232 (1985). 2866007 10.1192/bjp.147.3.221

[87] Abplanalp, S. J. et al. Understanding connections and boundaries between positive symptoms, negative symptoms, and role functioning among individuals with schizophrenia: a network psychometric approach. *JAMA Psychiatry* **79**, 1014–1022 (2022). 35976655 10.1001/jamapsychiatry.2022.2386 PMC9386606

[88] Fried, E. I. et al. Using network analysis to examine links between individual depressive symptoms, inflammatory markers, and covariates. *Psychol. Med.* **50**, 2682–2690 (2020). 31615595 10.1017/S0033291719002770

[89] Parksepp, M., Ljubajev, U., Taht, K. & Janno, S. Prevalence of neuroleptic-induced movement disorders: an 8-year follow-up study in chronic schizophrenia inpatients. *Nord J. Psychiatry* **70**, 498–502 (2016). 27093226 10.3109/08039488.2016.1164245

[90] Martino D., Karnik V., Osland S., Barnes T. R. E., Pringsheim T. M. Movement disorders associated with antipsychotic medication in people with schizophrenia: an overview of Cochrane reviews and meta-analysis. *Can. J. Psychiatry* 706743718777392 (2018). 10.1177/0706743718777392 PMC6299187 29758999

[91] Manschreck, T. C. & Ames, D. Neurologic features and psychopathology in schizophrenic disorders. *Biol. Psychiatry* **19**, 703–719 (1984). 6145453

[92] Heinrichs, D. W. & Buchanan, R. W. Significance and meaning of neurological signs in schizophrenia. *Am. J. Psychiatry* **145**, 11–18 (1988). 3276226 10.1176/ajp.145.1.11

[93] Altinok, DCA. et al. 3D-optical motion capturing examination of sensori- and psychomotor abnormalities in mental disorders: Progress and perspectives. *Neurosci Biobehav Rev.* **167**, 105917 (2024). 39389438 10.1016/j.neubiorev.2024.105917

[94] Galderisi, S. & Kaiser, S. EPA guidance on treatment of negative symptoms in schizophrenia. *Eur. Psychiatry* **64**, e21 (2021). 33726883 10.1192/j.eurpsy.2021.13 PMC8057437

[95] Wallwork, R. S., Fortgang, R., Hashimoto, R., Weinberger, D. R. & Dickinson, D. Searching for a consensus five-factor model of the Positive and Negative Syndrome Scale for schizophrenia. *Schizophr. Res.* **137**, 246–250 (2012). 22356801 10.1016/j.schres.2012.01.031 PMC3351536

