# Supplementary material for: Sensori- and psychomotor abnormalities, psychopathological symptoms and functionality in schizophrenia-spectrum disorders: a network analytic approach
Source: Schizophrenia (Heidelb). 2025 Feb 12;11(1):16. doi: 10.1038/s41537-024-00547-0 (PMC11821994; doi:10.1038/s41537-024-00547-0)
Supplement: Supplementary file 1 — Supplementary material [file 41537_2024_547_MOESM1_ESM.docx]

**Supplementary material:**

*Network stability:*

We employed bootstrapping as implemented in the *bootnet* package to evaluate edge stability as well as node (centrality) stability. Sufficient stability is a necessary requirement before interpreting the estimated networks. We calculated 95% confidence intervals and examined centrality stability of both expected influence and strength. Here, we calculated correlation stability coefficient defined by the proportion of participants which can be excluded from the original sample while keeping a correlation of at least 0.7 for centrality. The recommended correlation stability coefficient of 0.25 was exceeded in our analyses and consequently considered stable.

**Supplementary figure 1.** Edge stability as assessed by bootstrapping.

Edge-weights are sorted in increasing order (red). Grey areas represent 95% confidence intervals. Edge-edge relationships are depicted on the y- axis with labels.

**
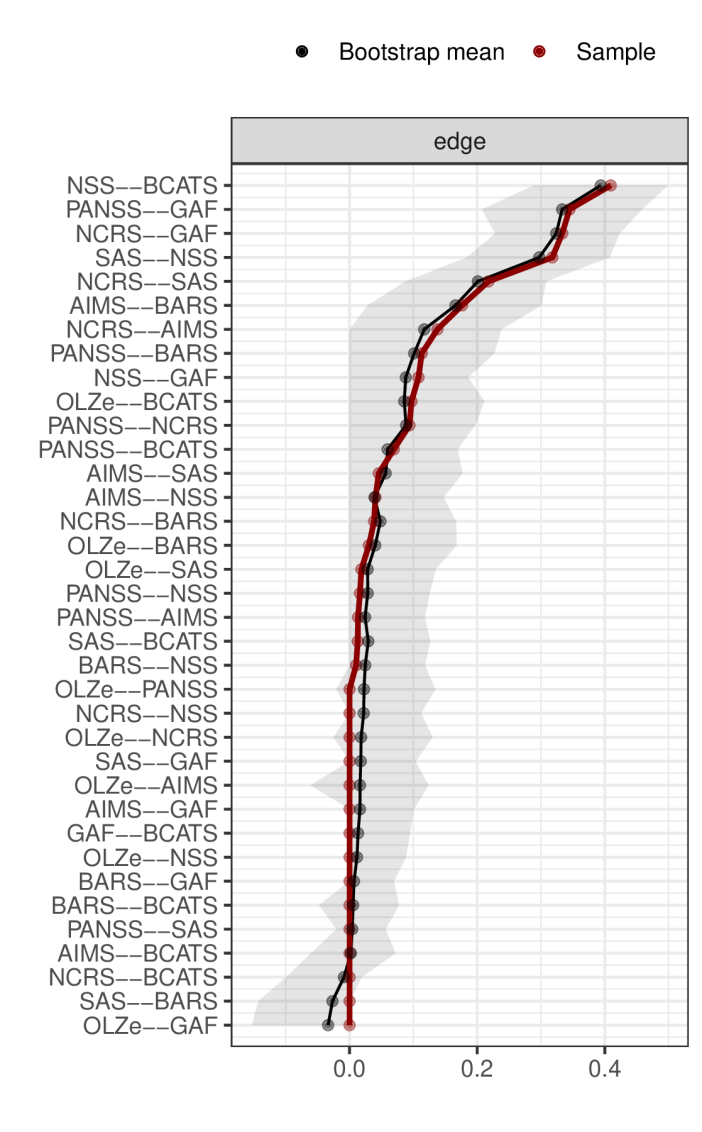
**

**Supplementary figure 2.** Case-dropping procedure to evaluate stability of expected influence and strength.

**
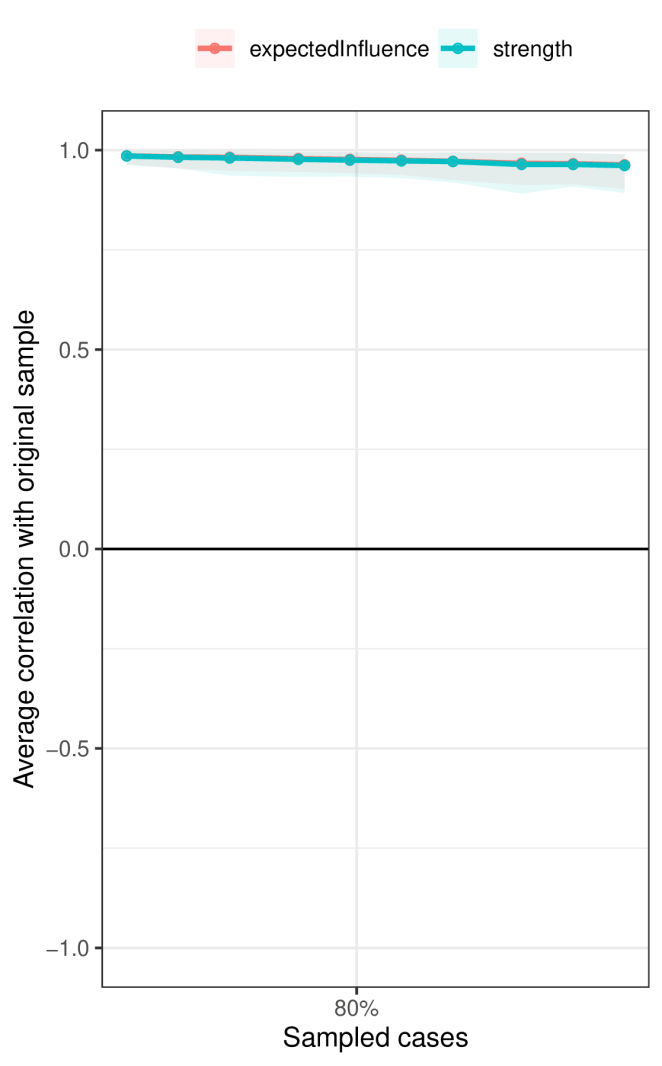
**

Correlations between expected influence and strength values and newly estimated expected influence in subgroups with decreasing participant percentages. Percentages of sampled participants are depicted on the x-axis. Average correlations are reported on the y-axis.

**Supplementary figure 3.** Centrality difference test (expected influence).


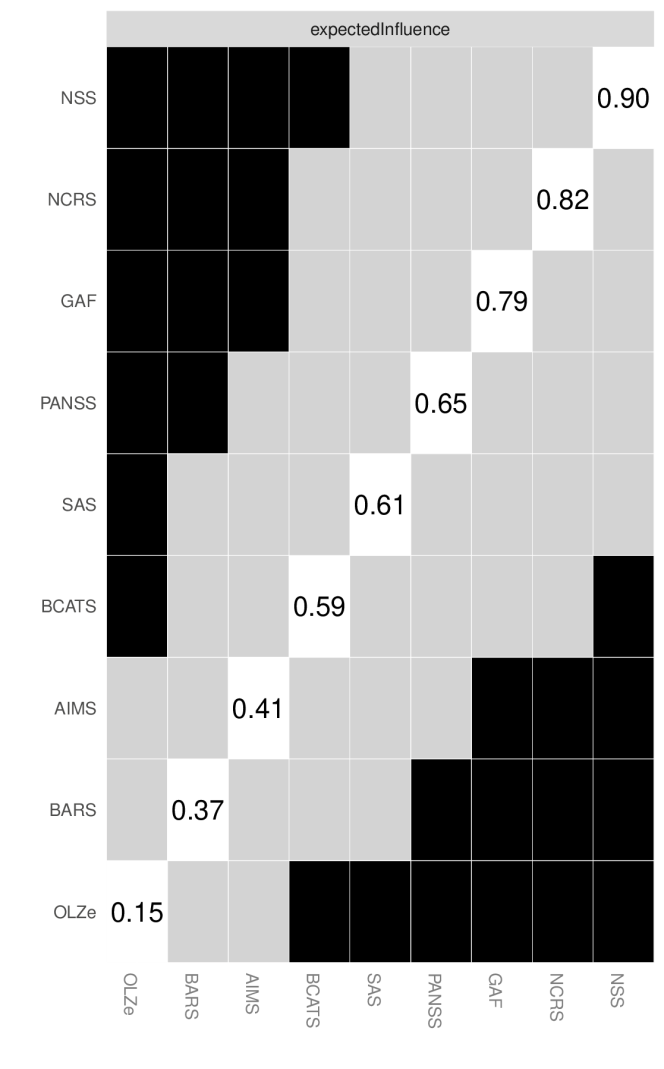


Black tiles represent significantly different centralities. Grey tiles represent non-significantly different centralities. Centrality of NSS, GAF and NCRS were significantly different from centrality of most other variables.

**Supplementary figure 4.** Centrality difference test (strength).


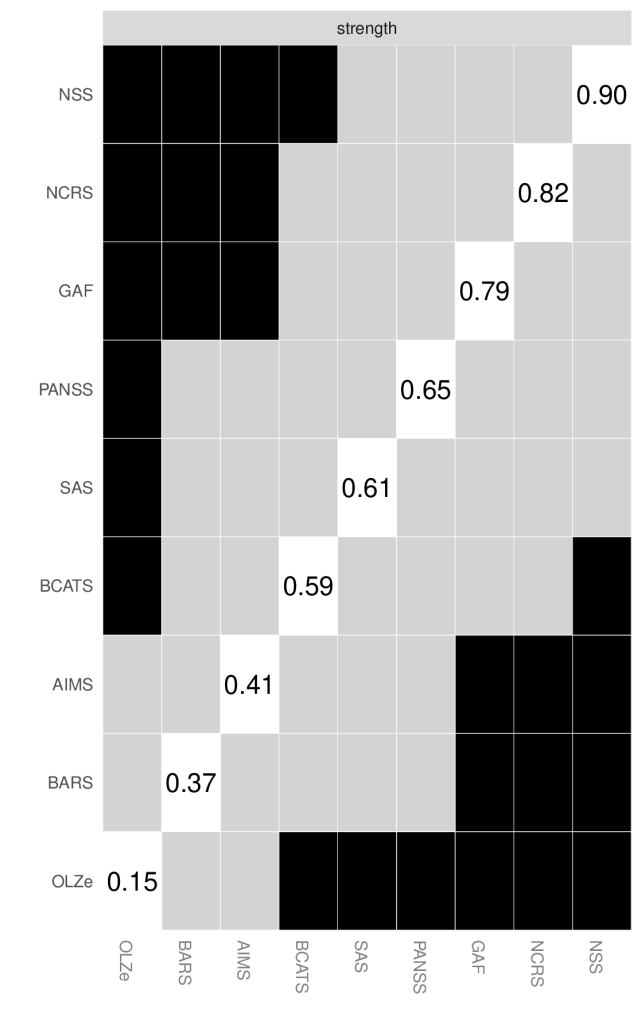


Black tiles represent significantly different centralities. Grey tiles represent non-significantly different centralities. Centrality of NSS, GAF and NCRS were significantly different from centrality of most other variables.

**Supplementary figure 5.** Edge-weight difference test.


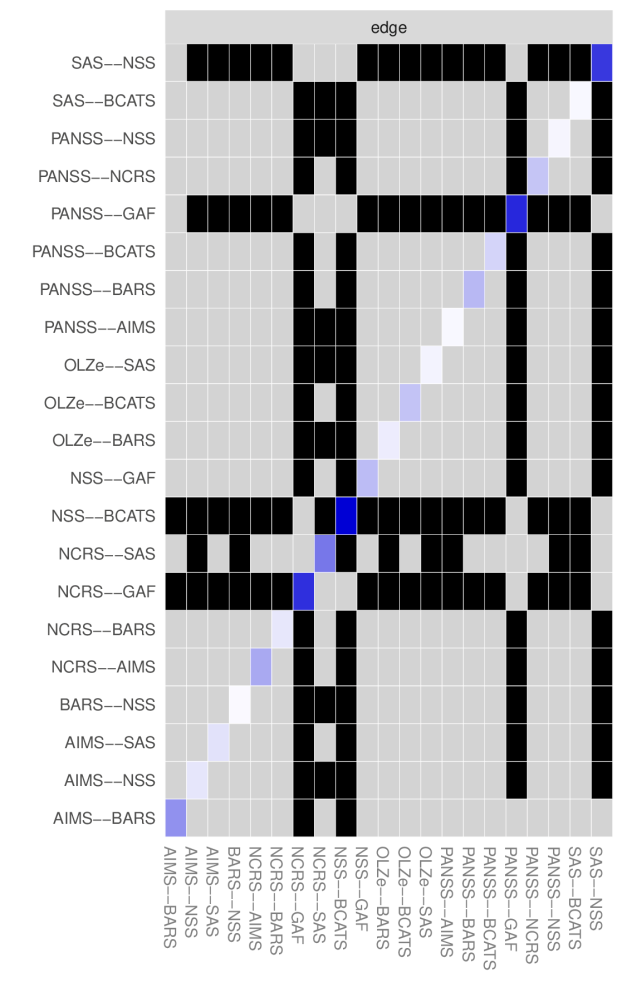


Black tiles represent significantly different edge-weights. Grey tiles represent non-significantly different edge-weights. Edge weight of NSS and B-CATS was significantly different from edge weight of most other variables, except edge-weight of SAS and NSS, PANSS and GAF as well as NCRS and GAF. Here, significance testing is not corrected for multiple testing.

**Supplementary figure 6.**


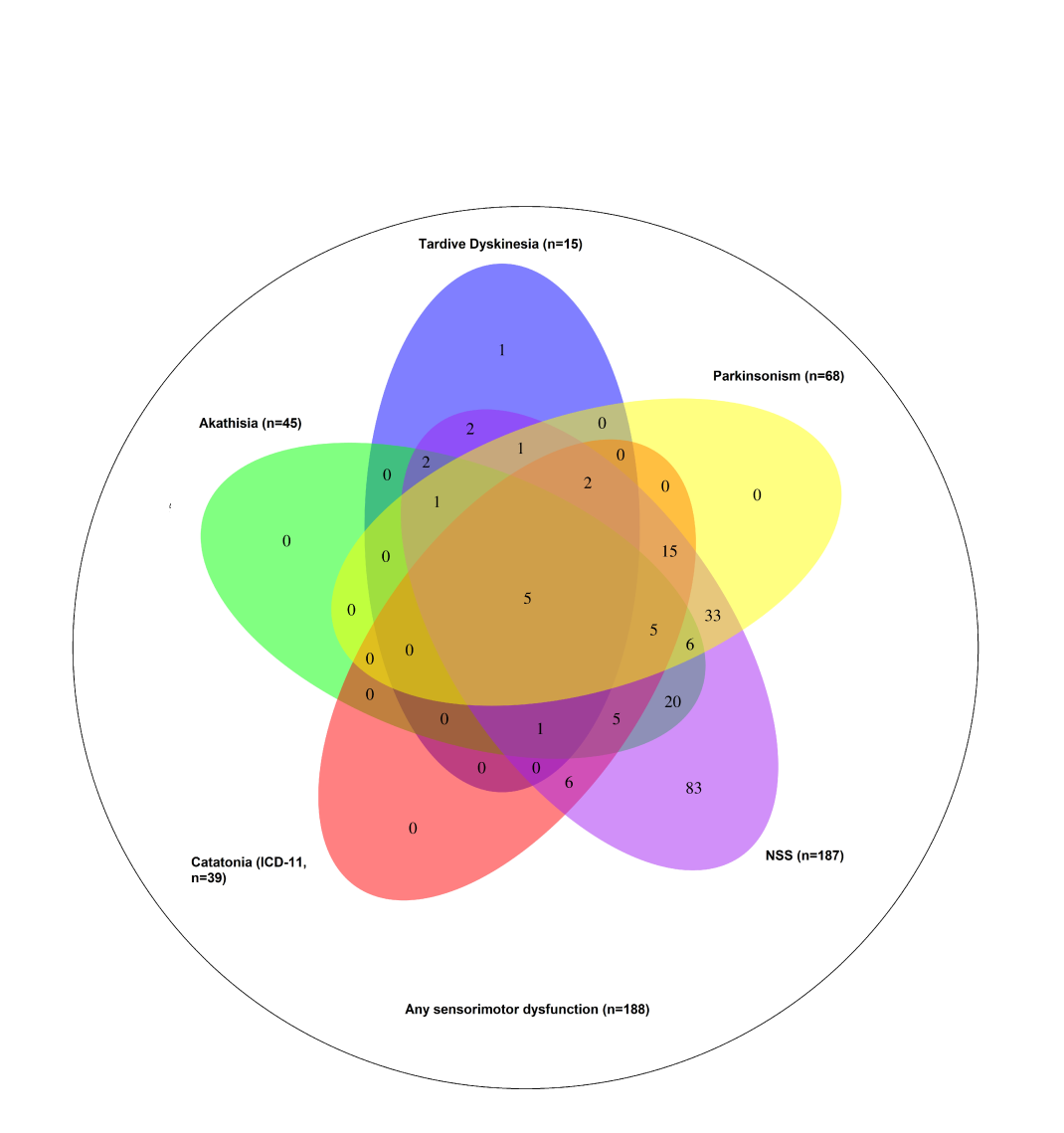


Venn diagram showing prevalence and overlap of sensorimotor and psychomotor abnormalities. Here catatonia was defined according to ICD-11 criteria.

**Network analysis of five motor nodes (NSS total score, BARS global score, NCRS total score, SAS total score, AIMS total score), PANSS total score, GAF score, B-CATS, OLZe, i.e. without regressing out the effects of age, sex and education:**

**Supplementary figure 7.**


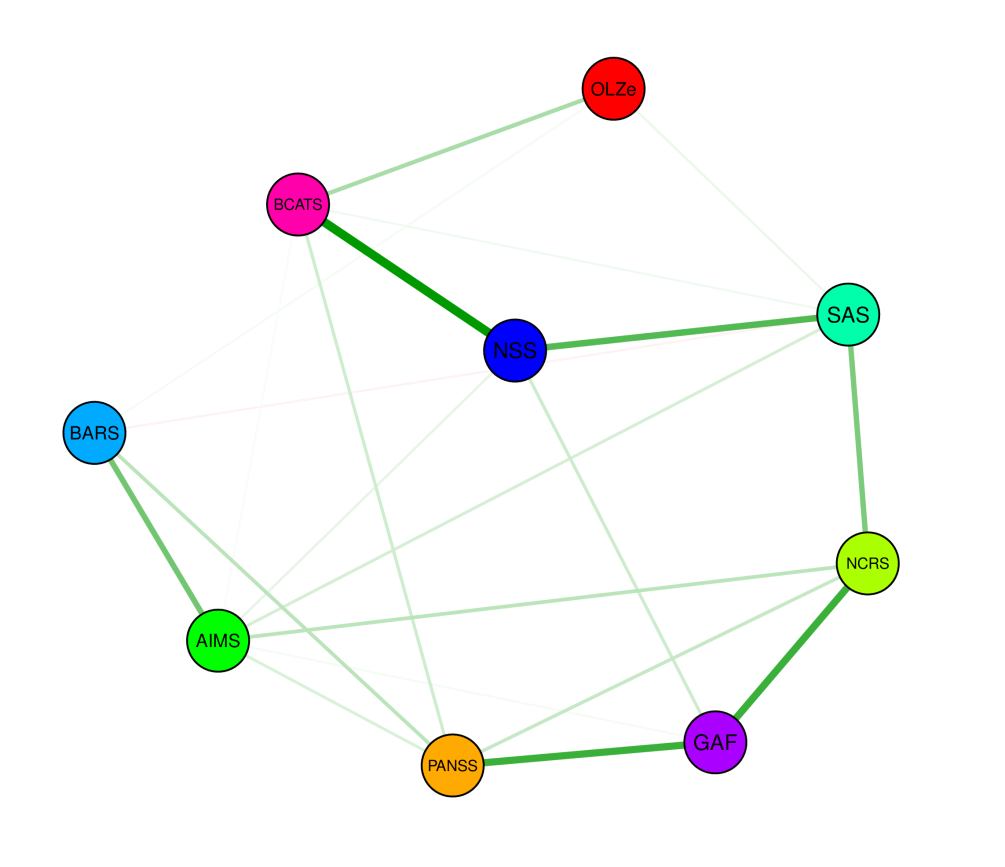

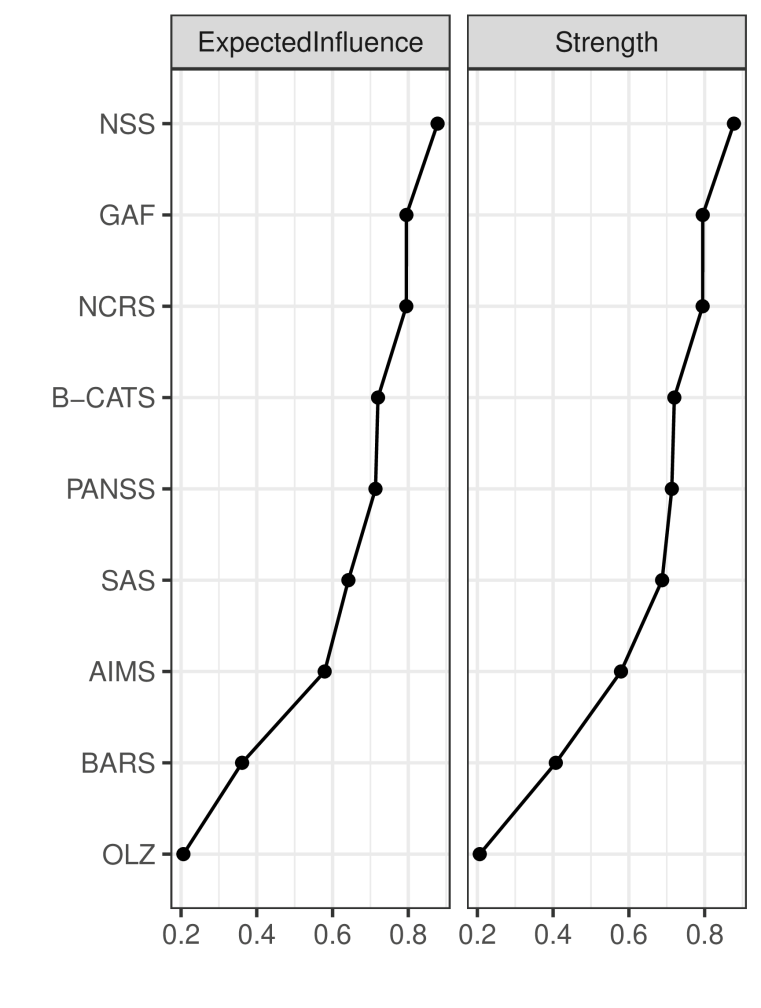


**Supplementary figure 8: Centrality stability (left) and edge stability (right).**


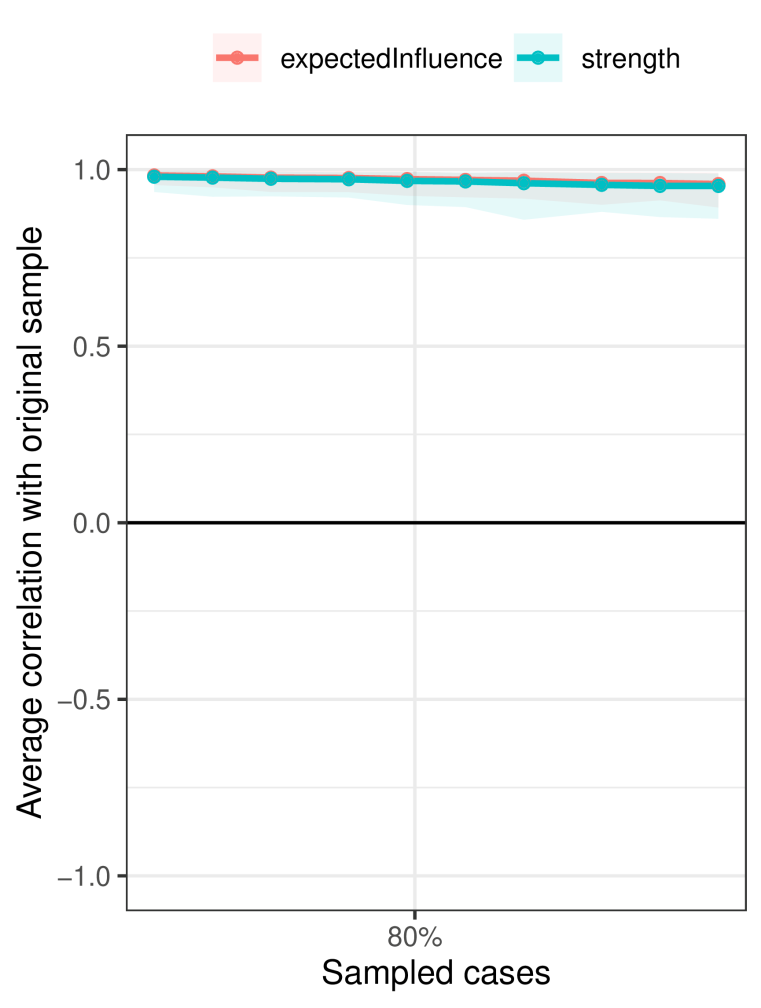

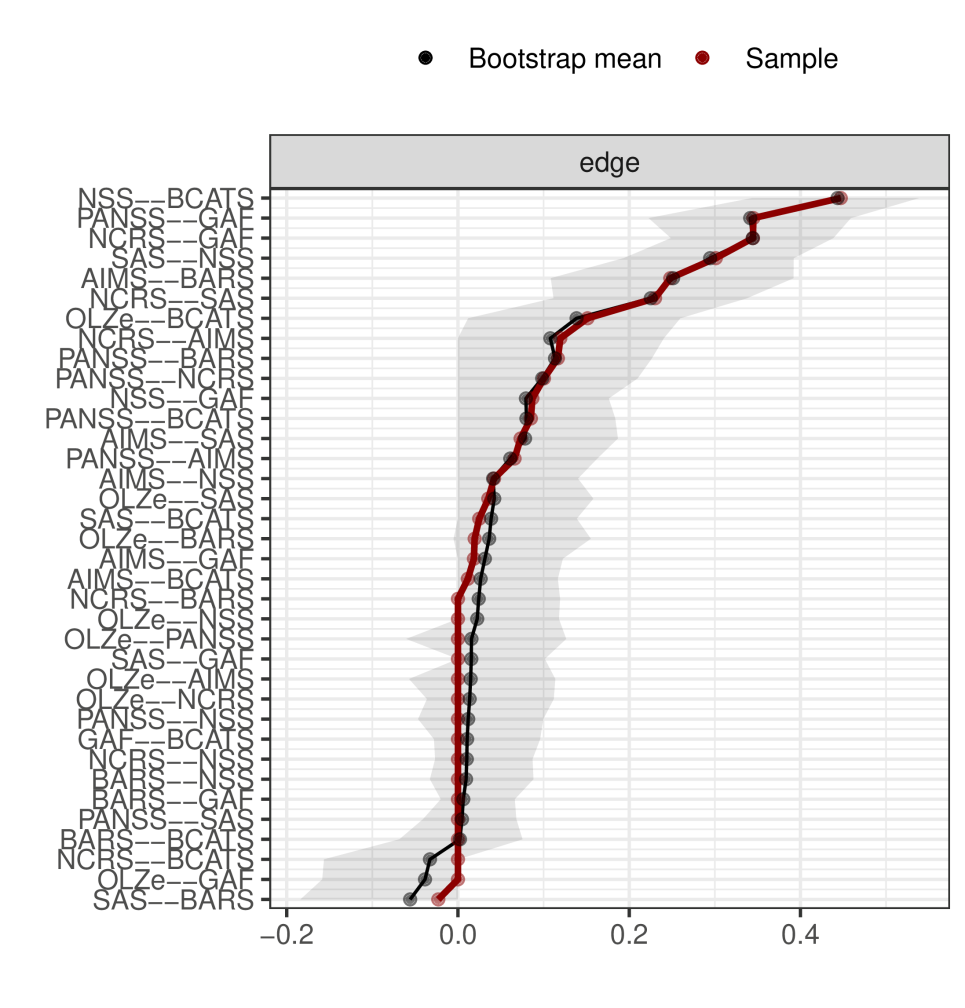


Case-dropping procedure to evaluate stability of expected influence and strength.

Edge-weights are sorted in increasing order (red). Grey areas represent 95% confidence intervals. Edge-edge relationships are depicted on the y- axis with labels.

**Supplementary figure 9: Centrality difference test of expected influence (left) and strength (right).**


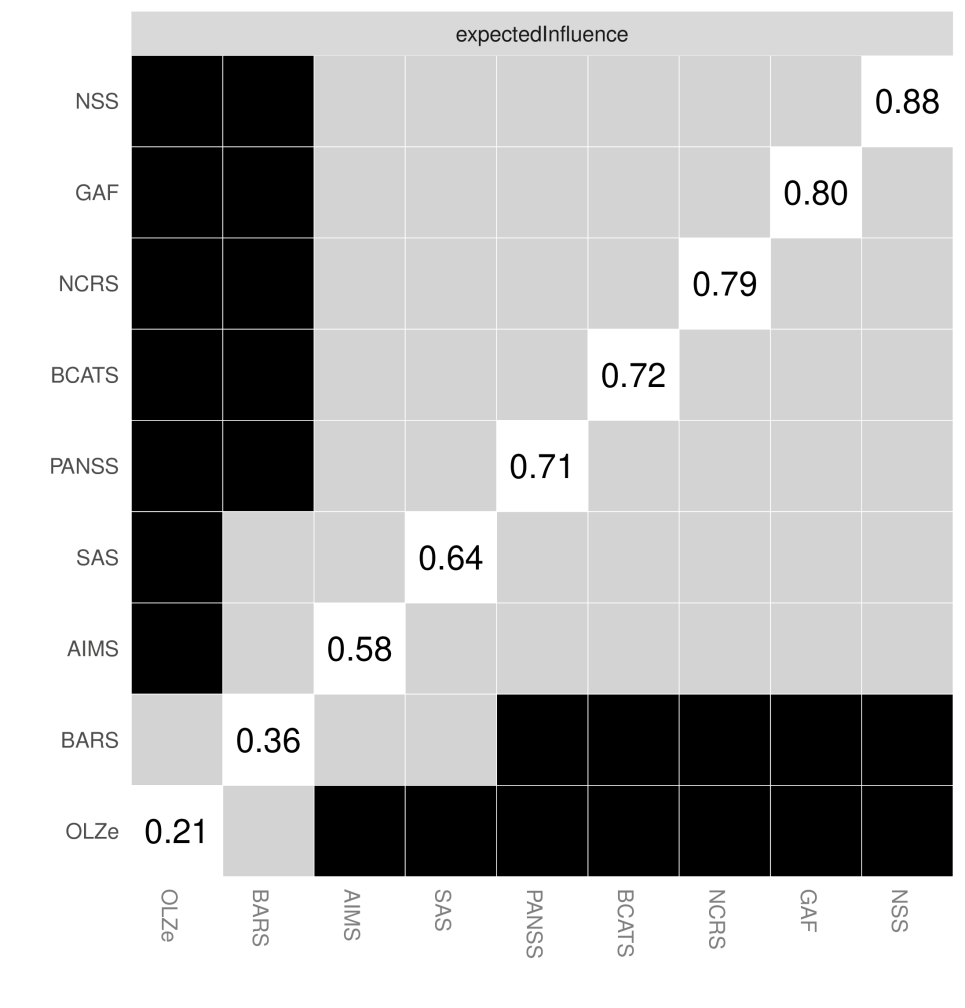

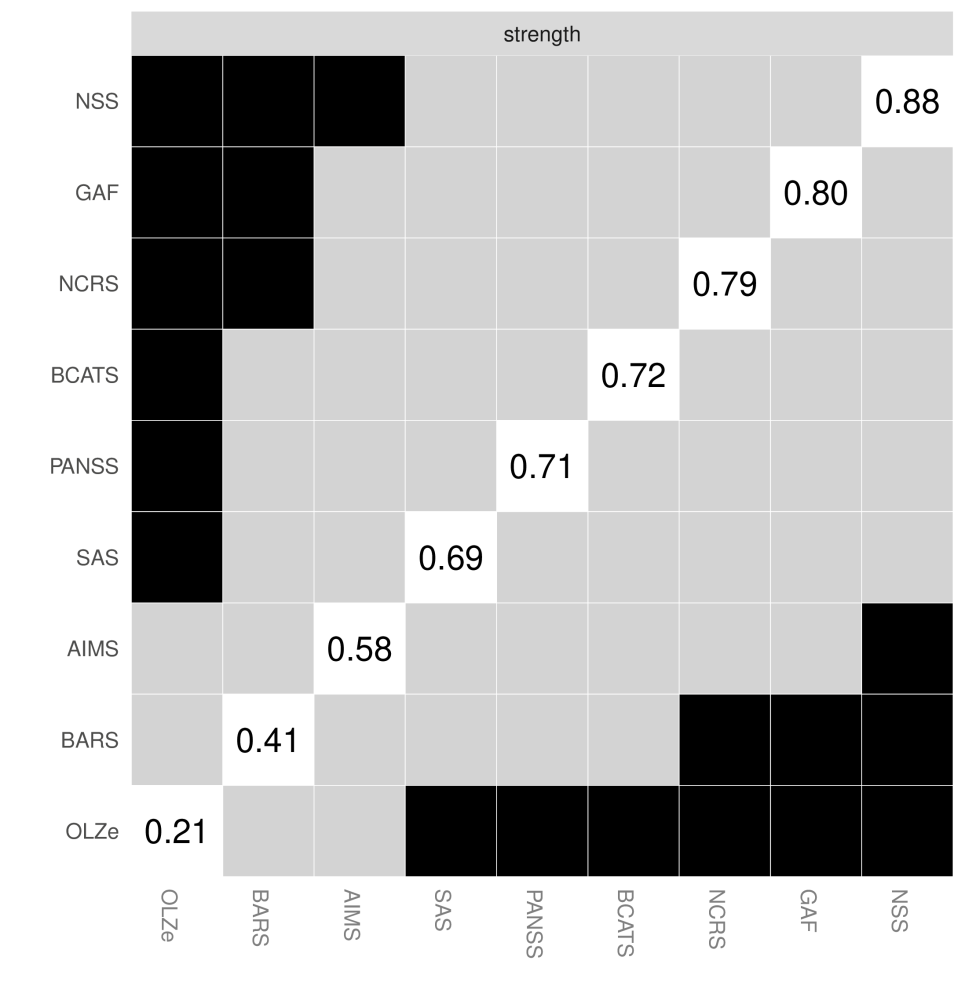


Black tiles represent significantly different centralities. Grey tiles represent non-significantly different centralities.

**Supplementary figure 10:** Edge-weight difference test.


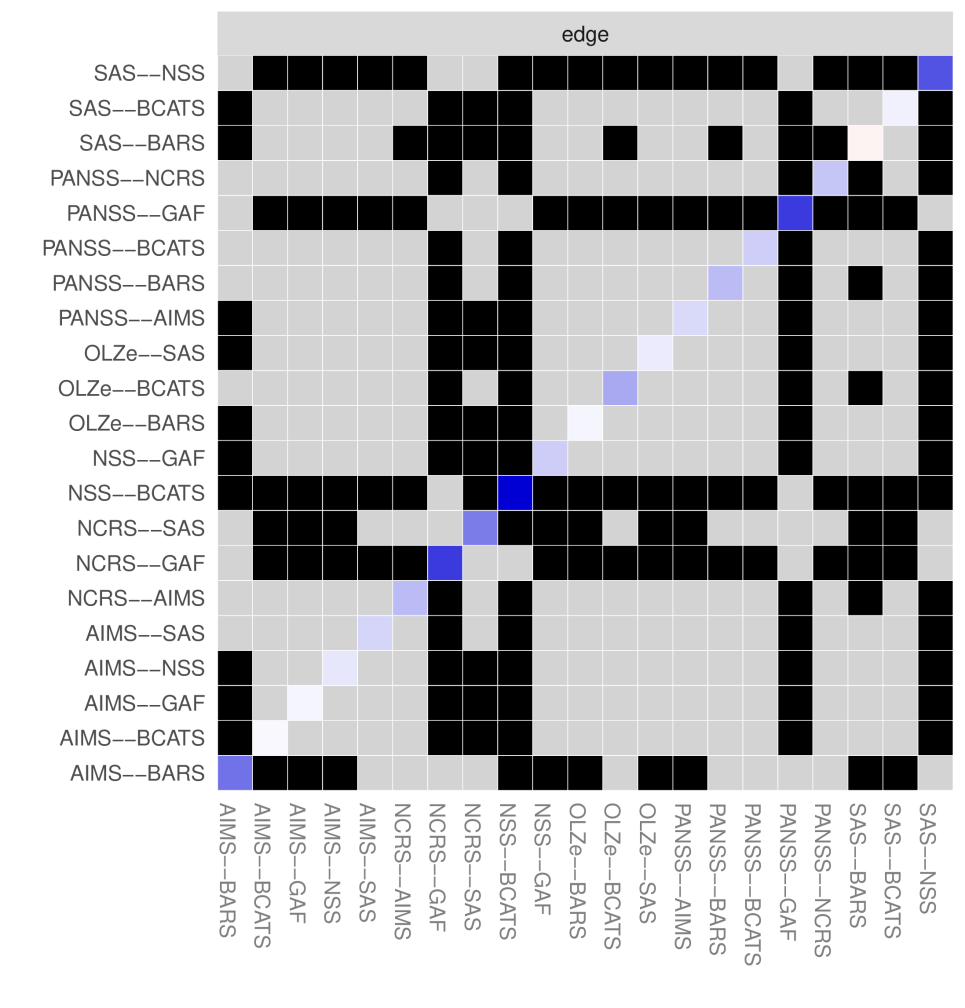


Black tiles represent significantly different edge-weights. Grey tiles represent non-significantly different edge-weights.

**Network analysis of five motor nodes after regressing out age, sex and education:**

**Supplementary figure 11:**


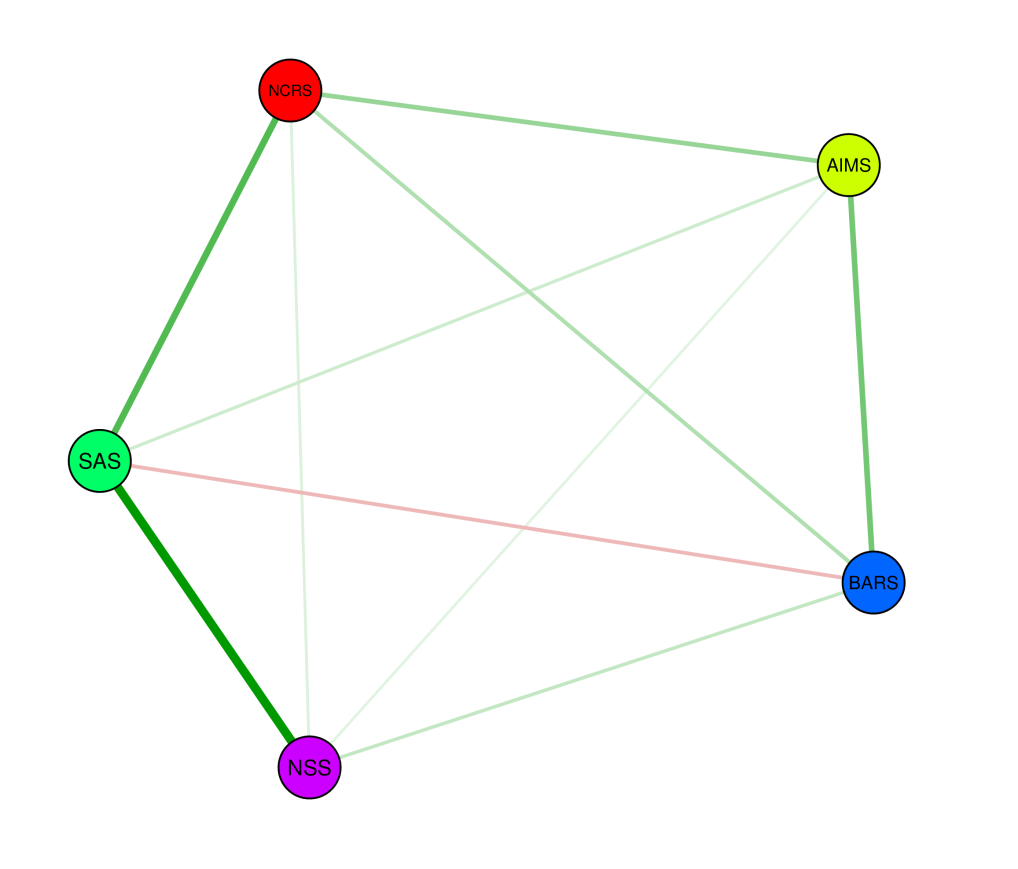

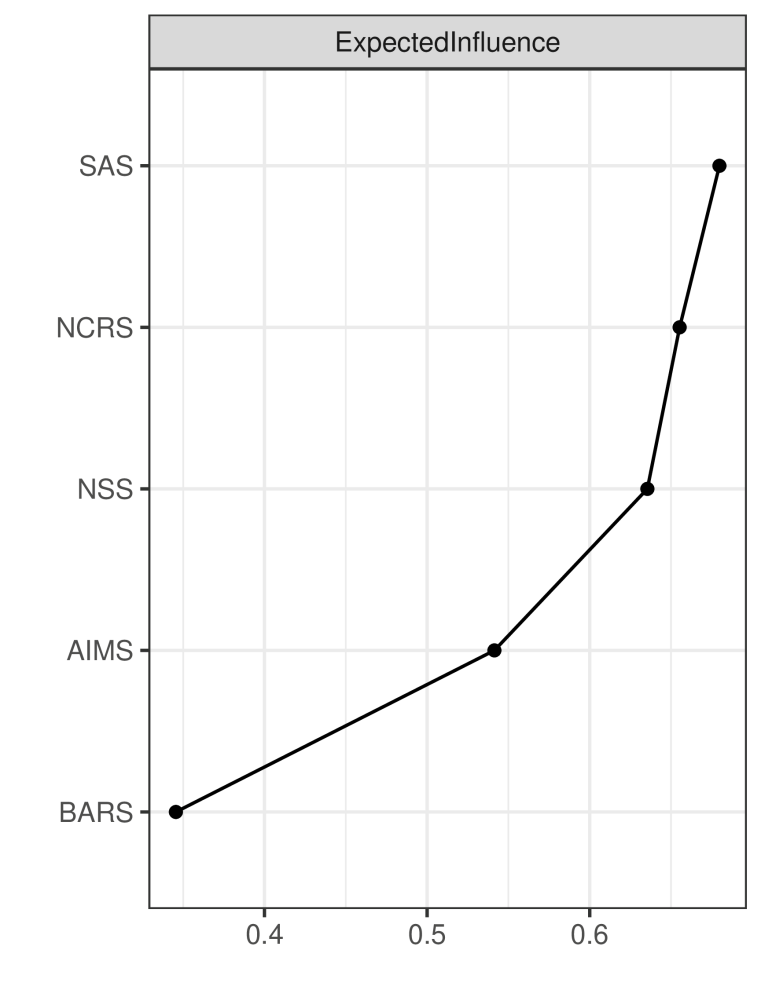


**Supplementary figure 12:** Edge stability as assessed by bootstrapping (left). Centrality stability of expected influence (right).


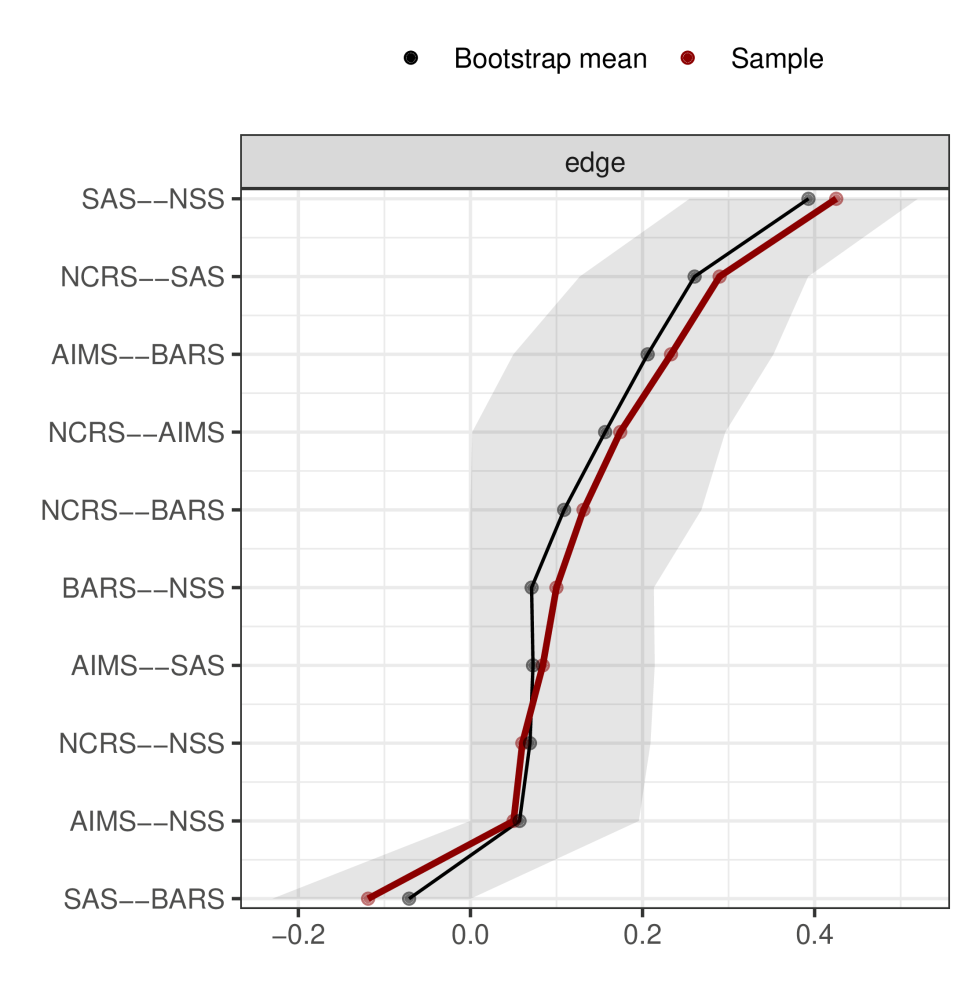

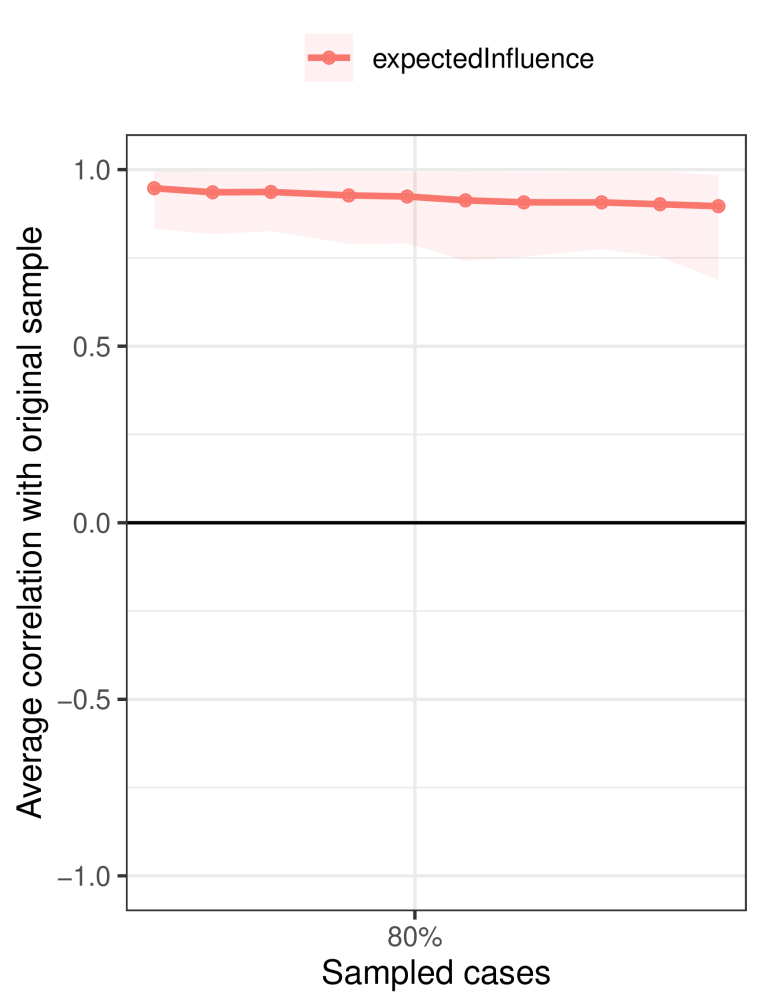


Case-dropping procedure to evaluate stability of expected influence

Edge-weights are sorted in increasing order (red). Grey areas represent 95% confidence intervals. Edge-edge relationships are depicted on the y- axis with labels.

**Supplementary figure 13:** Edge-weight difference test (left). Centrality difference test (expected influence).


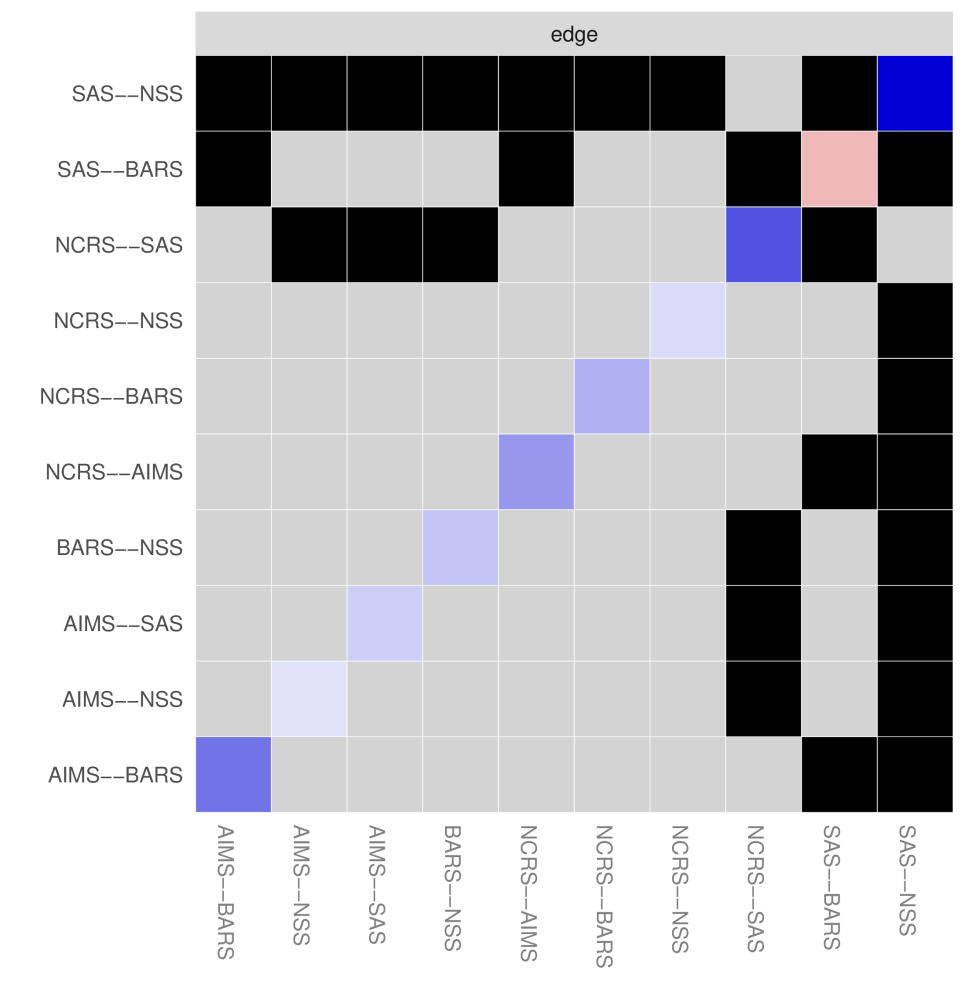

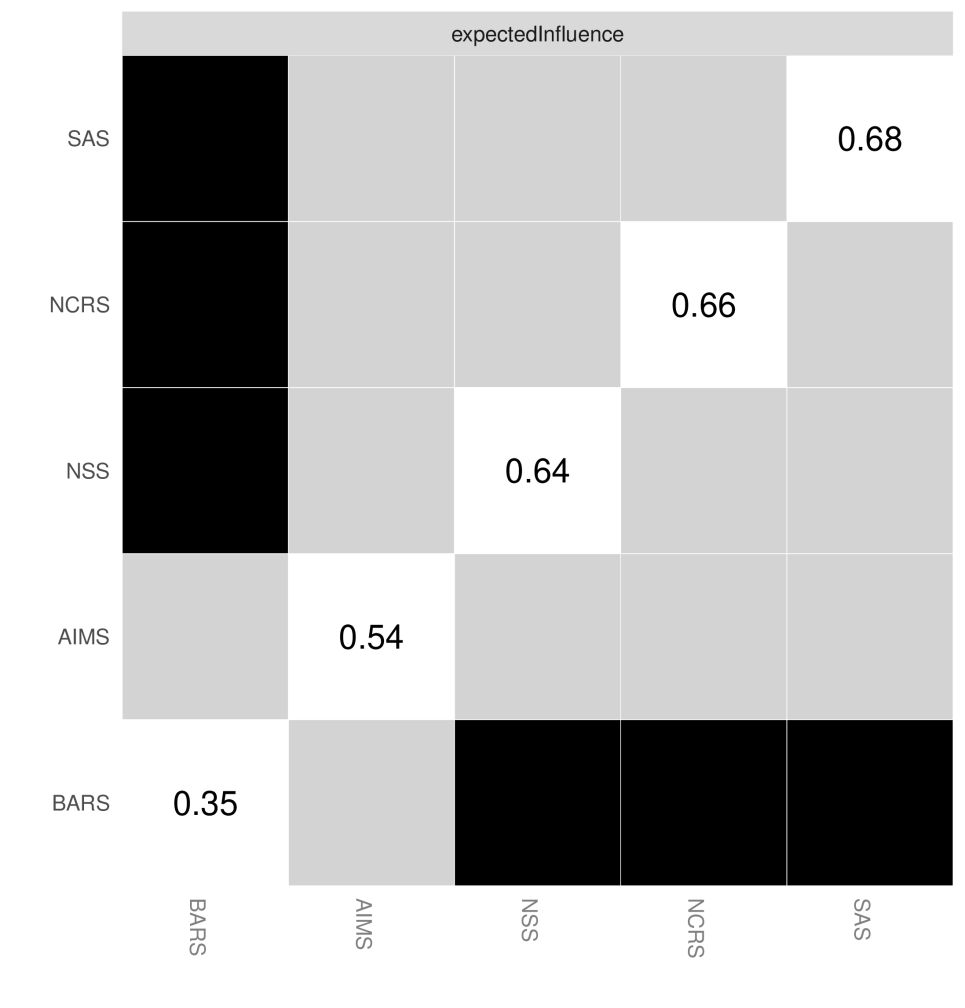


Black tiles represent significantly different centralities. Grey tiles represent non-significantly different centralities.

Black tiles represent significantly different edge-weights. Grey tiles represent non-significantly different edge-weights.

**Network analysis of five motor nodes:**

**Supplementary figure 14:**


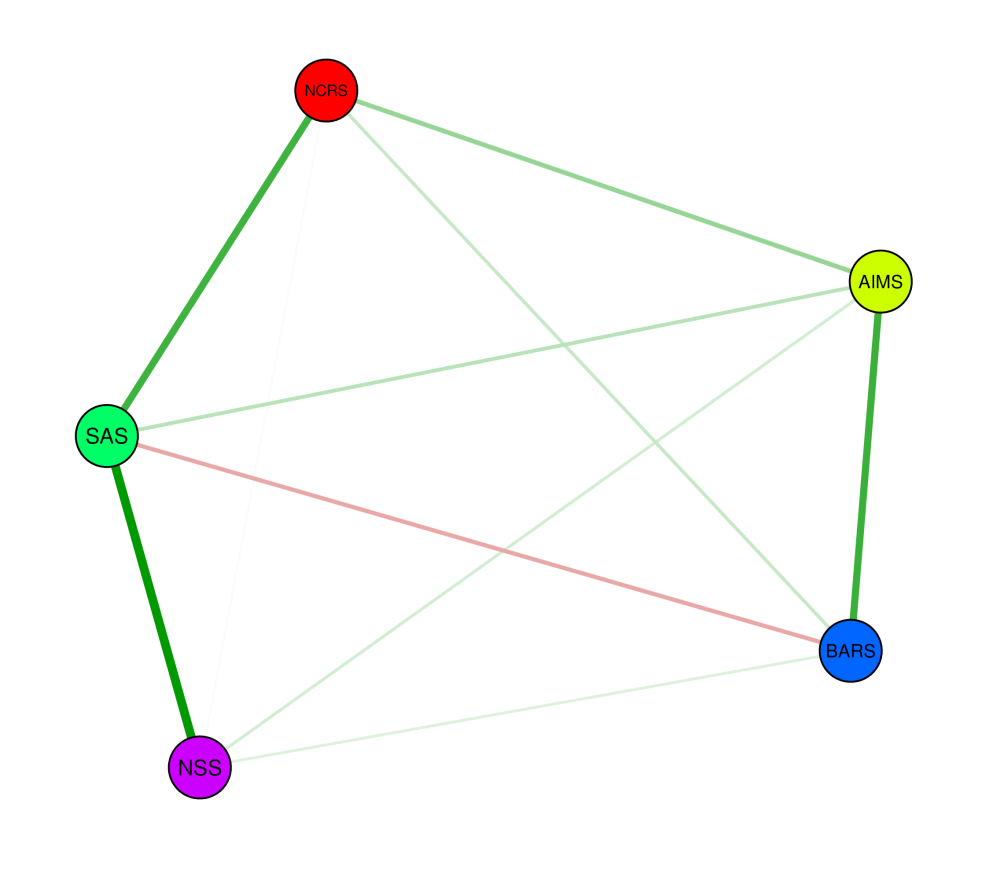

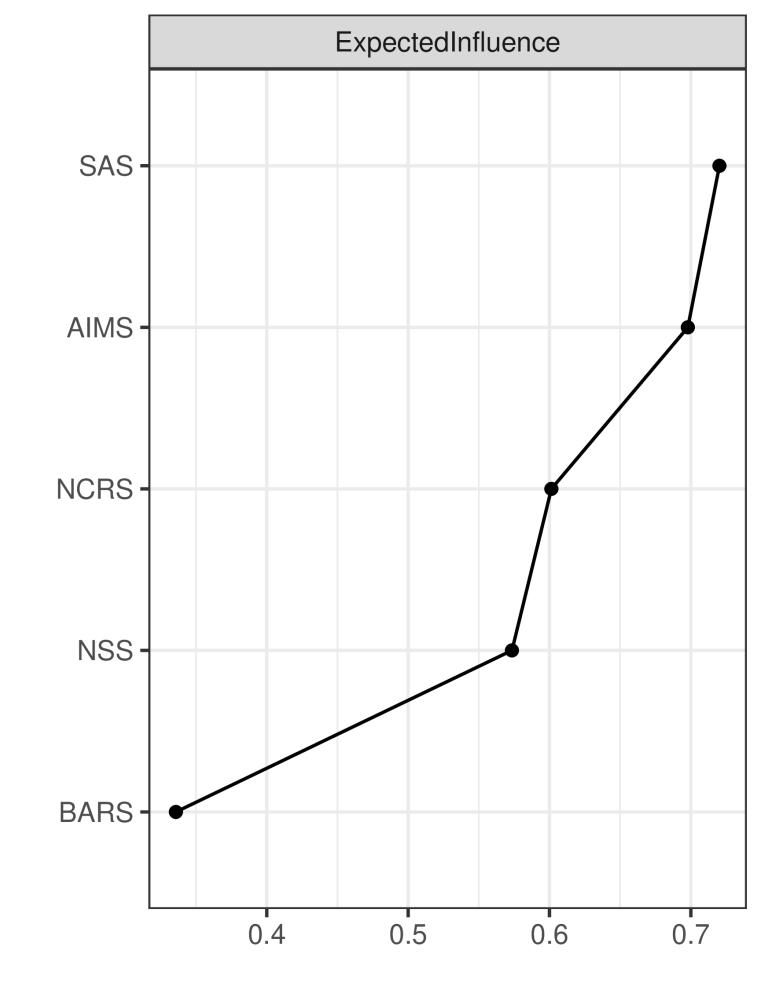


**Supplementary figure 15:** Centrality stability of expected influence (left). Edge stability as assessed by bootstrapping (right).


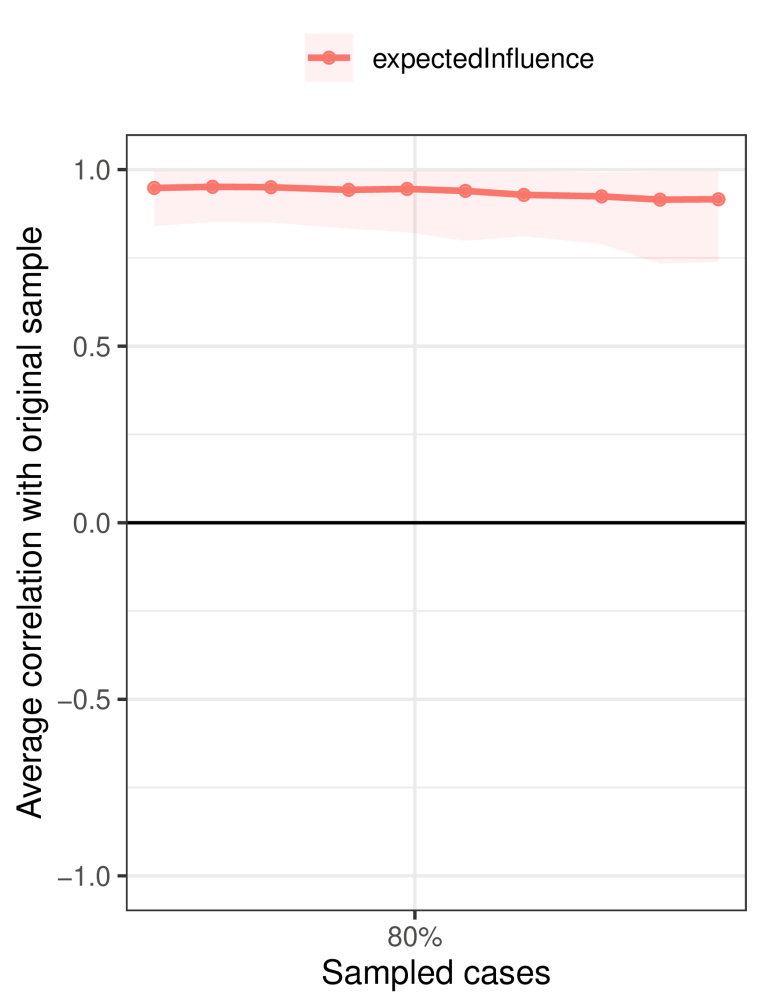

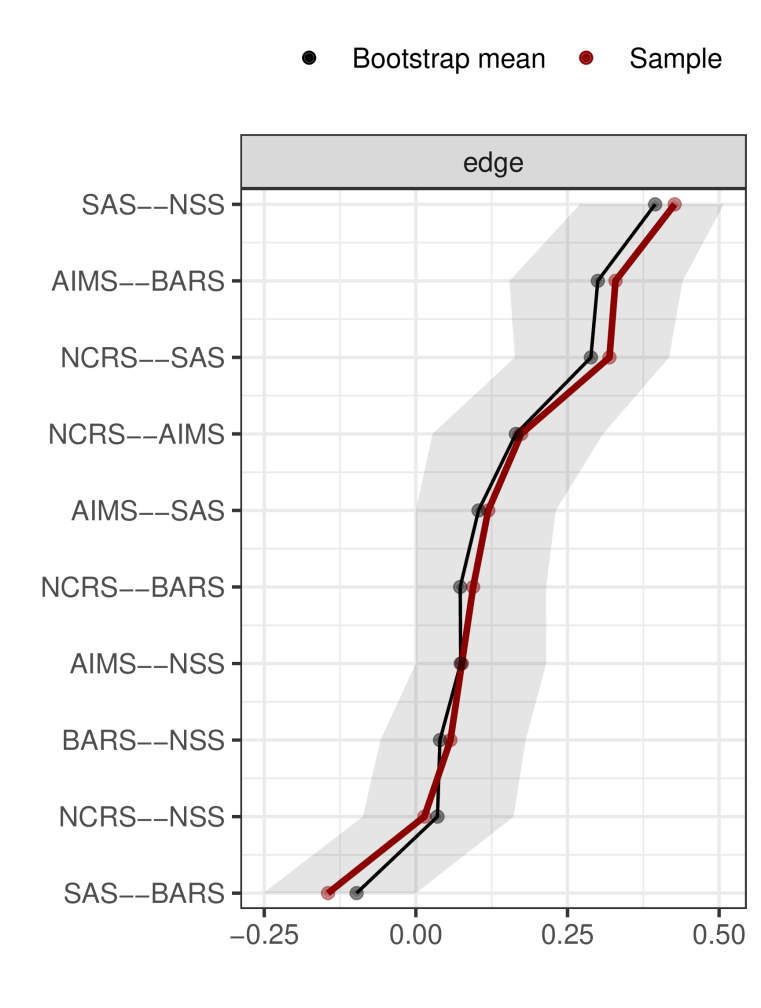


Case-dropping procedure to evaluate stability of expected influence.

Edge-weights are sorted in increasing order (red). Grey areas represent 95% confidence intervals. Edge-edge relationships are depicted on the y- axis with labels.

**Supplementary figure 16:** Centrality difference test for expected influence (left). Edge-weight difference test (right).


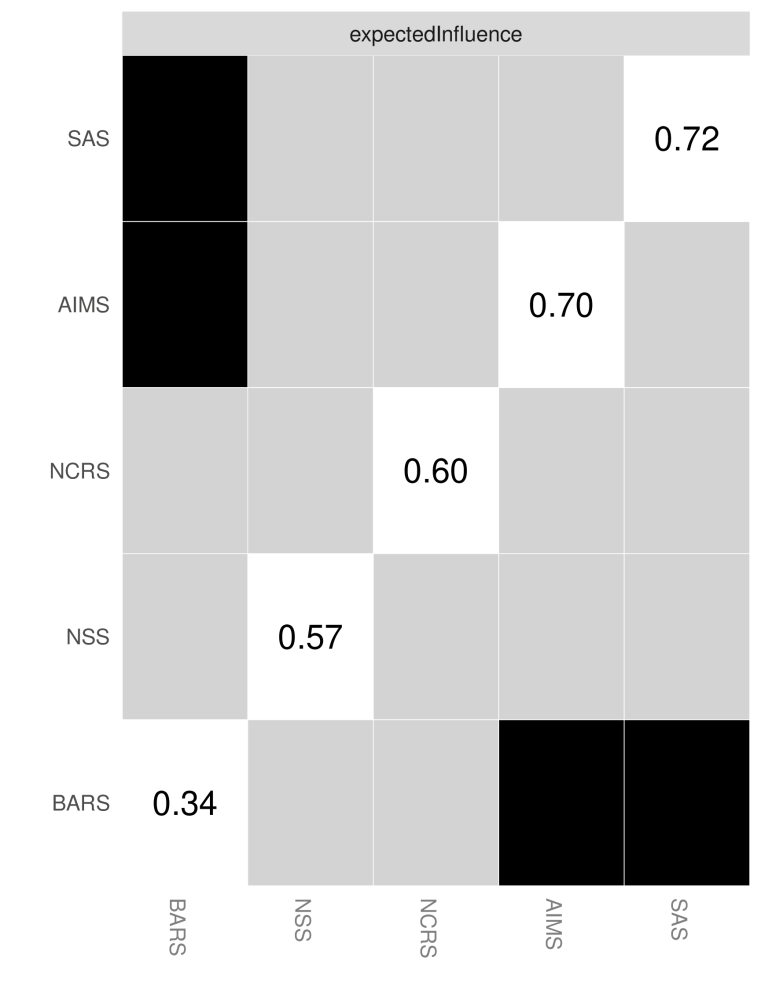

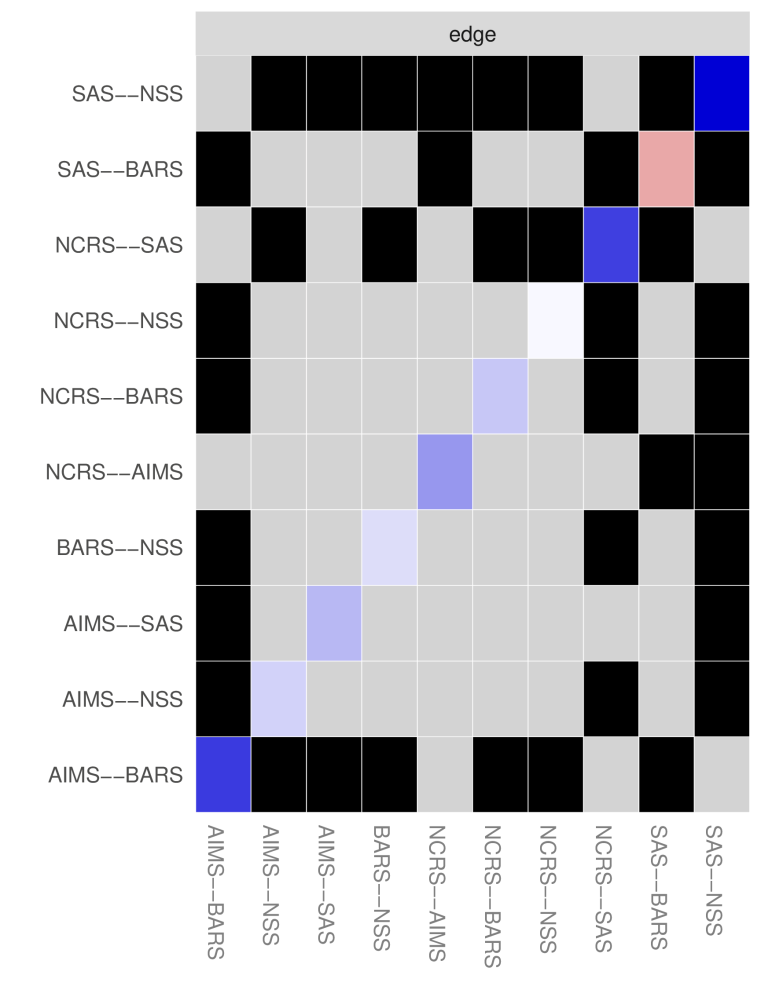


Black tiles represent significantly different edge-weights. Grey tiles represent non-significantly different edge-weights.

Black tiles represent significantly different centralities. Grey tiles represent non-significantly different centralities.

**Nodewise predictability stability:**

**Supplementary figure 17:**


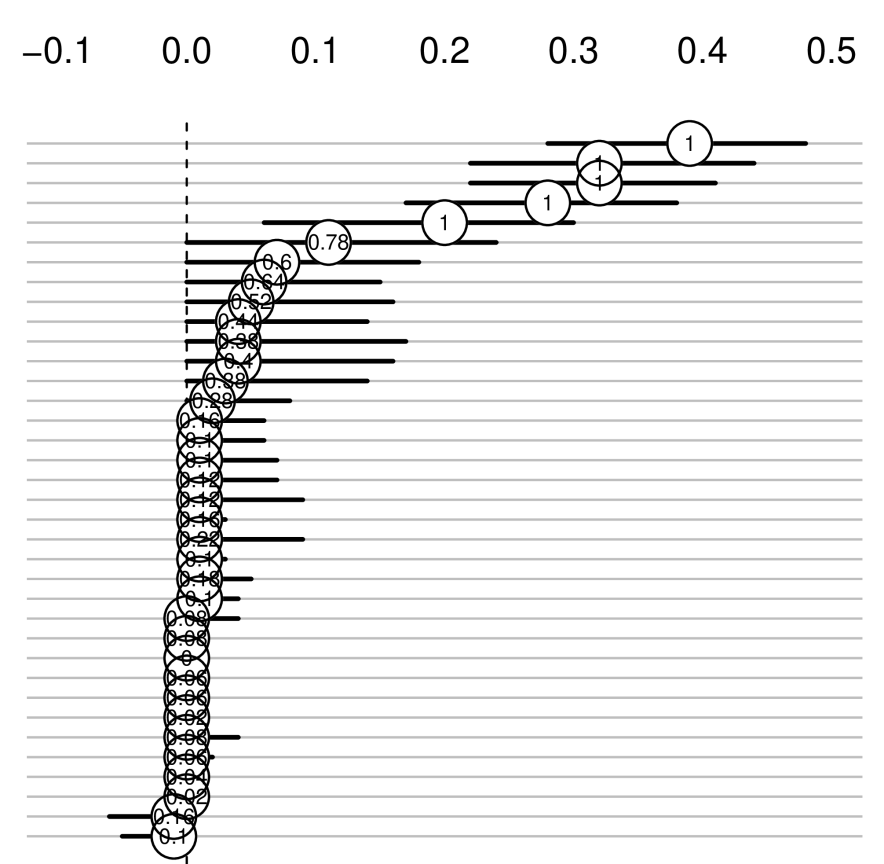


The horizontal lines show the 5% and 95% quantiles of the bootstrapped sampling distributions. Circled number: proportion of bootstrap samples in which a parameter has been estimated to be nonzero. Placing of the circled number indicates the location of the mean of the sampling distribution on the x-axis.

**Supplementary table 1.** Clinical and demographic variables in schizophrenia spectrum disorders (SSD; *n*=192), separately for both included cohorts.

|  | **Cohort #1 (n=129)** |  | **Cohort #2 (n=63)** |  |  |
| --- | --- | --- | --- | --- | --- |
|  | **Mean** | **SD** | **Mean** | **SD** | ***W/X²*** |
| Age | 38.35 | 11.58 | 36.51 | 13.24 | 0.349 |
| Sex (male/female) | 72/57 | - | 47/16 | - | **0.012** |
| Education | 13.02 | 2.85 | 13.62 | 3.20 | 0.212 |
| OLZe | 17.52 | 10.61 | 13.48 | 9.24 | **0.008** |
| PANSS total score | 67.20 | 21.12 | 66.63 | 16.36 | 0.839 |
| GAF score* | 50.63 | 17.18 | 73.86 | 12.80 | **<0.001** |
| NSS total | 19.80 | 8.75 | 21.78 | 9.11 | 0.155 |
| NCRS total score | 2.95 | 3.29 | 6.40 | 4.73 | **<0.001** |
| SAS total score | 2.95 | 2.55 | 3.98 | 3.33 | **0.033** |
| AIMS total score | 1.05 | 2.41 | 1.17 | 2.37 | 0.743 |
| BARS global score | 0.91 | 1.30 | 0.52 | 0.88 | **0.017** |
| TMT-B | 115.53 | 64.39 | 102.41 | 66.31 | 0.196 |
| DSST* | 65.69 | 18.68 | 57.94 | 19.75 | **0.011** |
| CF* | 15.92 | 4.68 | 15.44 | 4.12 | 0.469 |
|  | Skewness | Kurtosis | Skewness | Kurtosis |  |
| Age | 0.22 | 2.10 | 0.37 | 1.86 | - |
| Sex (male/female) | - | - | - | - | - |
| Education | 0.05 | 5.11 | 0.63 | 2.73 | - |
| OLZe | 0.64 | 3.36 | 0.35 | 2.23 | - |
| PANSS total score | 0.61 | 3.24 | 0.05 | 2.31 | - |
| GAF score* | 0.47 | 2.62 | -0.43 | 3.27 | - |
| NSS total | 0.70 | 2.84 | 1.05 | 3.76 | - |
| NCRS total score | 1.32 | 4.79 | 1.29 | 5.77 | - |
| SAS total score | 1.31 | 5.05 | 1.14 | 3.71 | - |
| AIMS total score | 2.99 | 11.97 | 2.72 | 10.06 | - |
| BARS global score | 1.36 | 3.95 | 1.52 | 4.14 | - |
| TMT-B | 1.31 | 4.48 | 2.35 | 8.54 | - |
| DSST* | -0.19 | 2.68 | 0.41 | 4.91 | - |
| CF* | -0.46 | 2.69 | -0.34 | 3.99 | - |

Data are mean ± standard deviation and Welch or Chi-square test and uncorrected *p* values

Abbreviations: *SD* Standard Deviation*, PANSS* Positive and Negative Symptoms Scale, *GAF* Global Assessment of Functioning Scale, *NSS* Neurological Soft Signs, *NCRS* Northoff Catatonia Rating Scale, *SAS* Simpson Angus Scale, *AIMS* Abnormal Involuntary Movement Scale, *BARS* Barnes Akathisia Rating Scale, *TMT-B* Trail Making Test B, *DSST* Digit Symbol Substitution Test, *CF* Category Fluency.

*reverse-coded values

**Supplementary table 2.** Spearman correlation between B-CATS and motor abnormalities in SSD (*n*=192).

| Variable 1 | Variable 2 | *ρ* | *p* |
| --- | --- | --- | --- |
| B-CATS | NSS | **0.571** | **<0.001** |
| B-CATS | AIMS | **0.155** | **0.031** |
| B-CATS | SAS | **0.284** | **<0.001** |
| B-CATS | BARS | 0.038 | 0.599 |
| B-CATS | NCRS | 0.060 | 0.408 |

Data are Spearman correlation results, uncorrected p-values.

Abbreviations: *ρ* Spearman´s Rho*, NSS* Neurological Soft Signs, *NCRS* Northoff Catatonia Rating Scale, *SAS* Simpson Angus Scale, *AIMS* Abnormal Involuntary Movement Scale, *BARS* Barnes Akathisia Rating Scale, *B-CATS* Brief Cognitive Assessment Tool for Schizophrenia.

**Five motor nodes, PANSS subscores (Positive, Negative, General), GAF score, B-CATS, OLZe after regressing out age, sex and education:**

**Supplementary figure 18:**


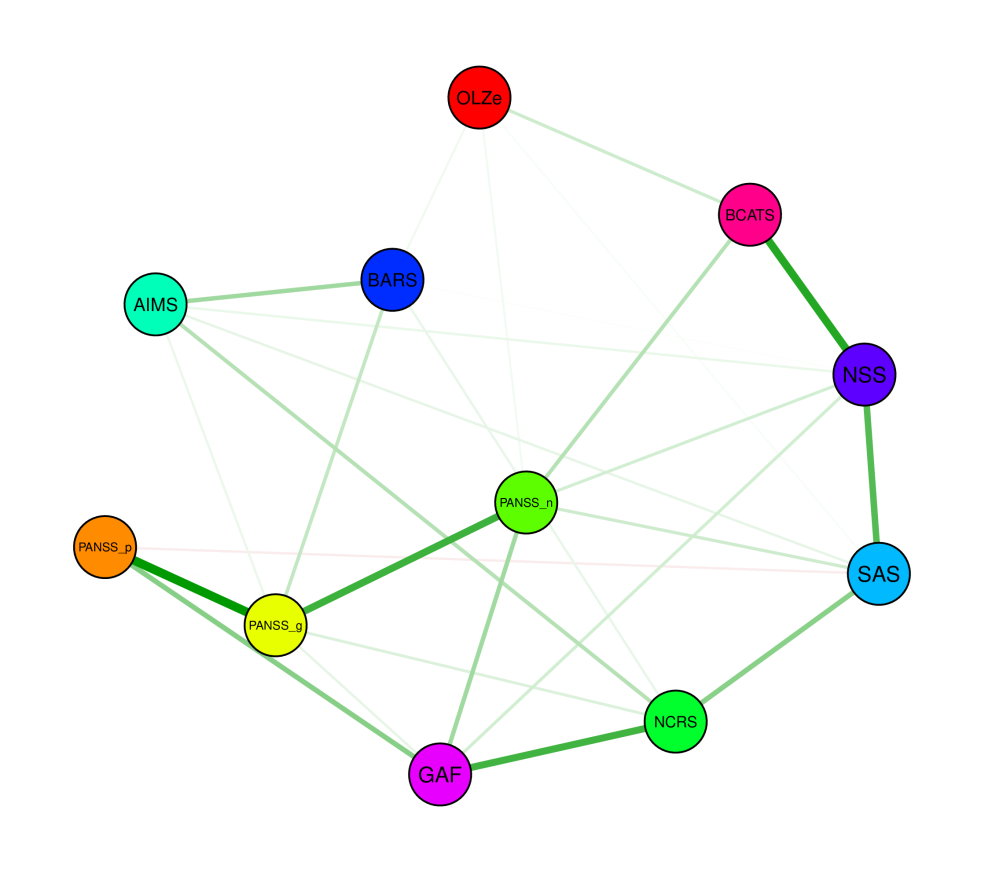

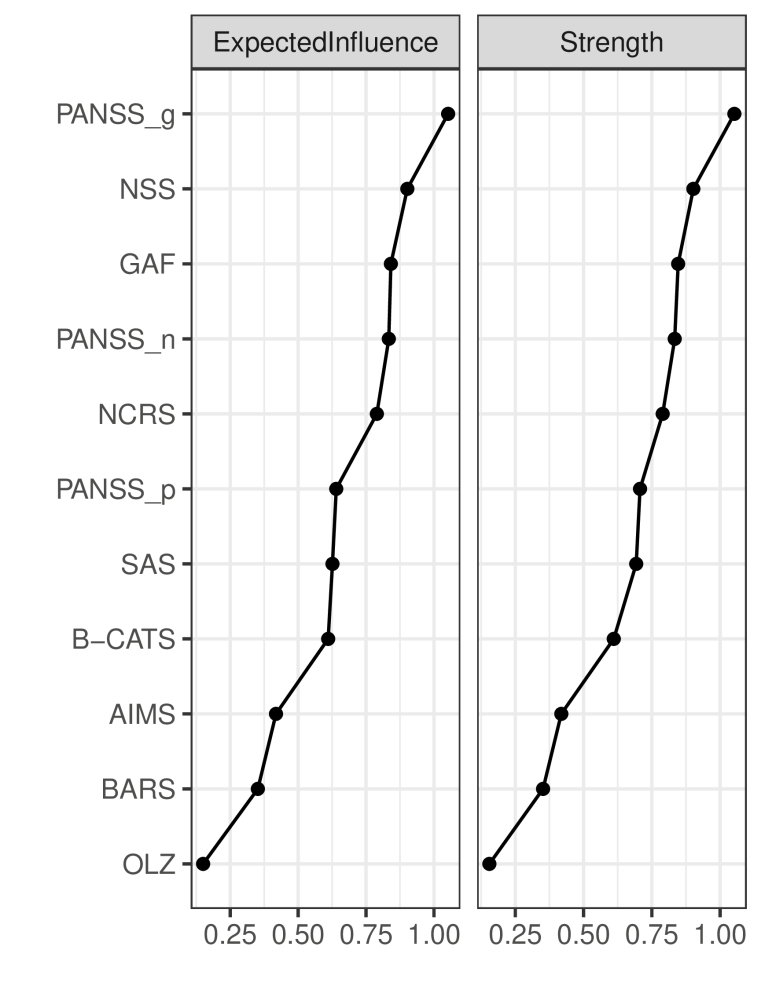


**Supplementary figure 19:** Centrality stability of expected influence and strength (left). Edge stability as assessed by bootstrapping (right).


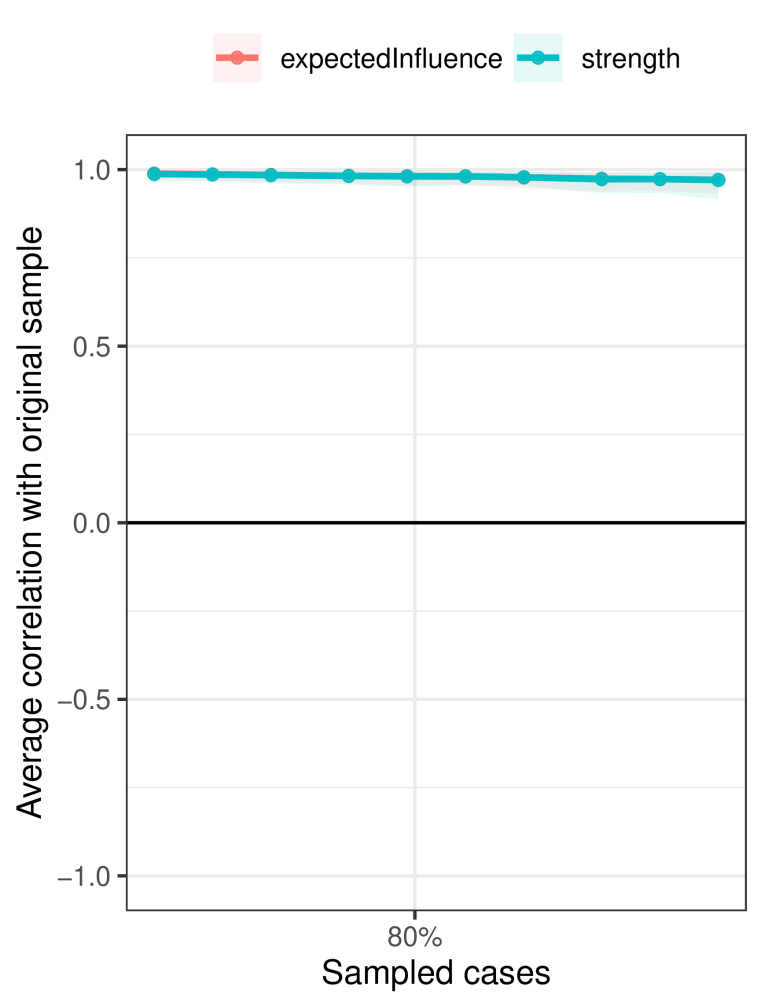

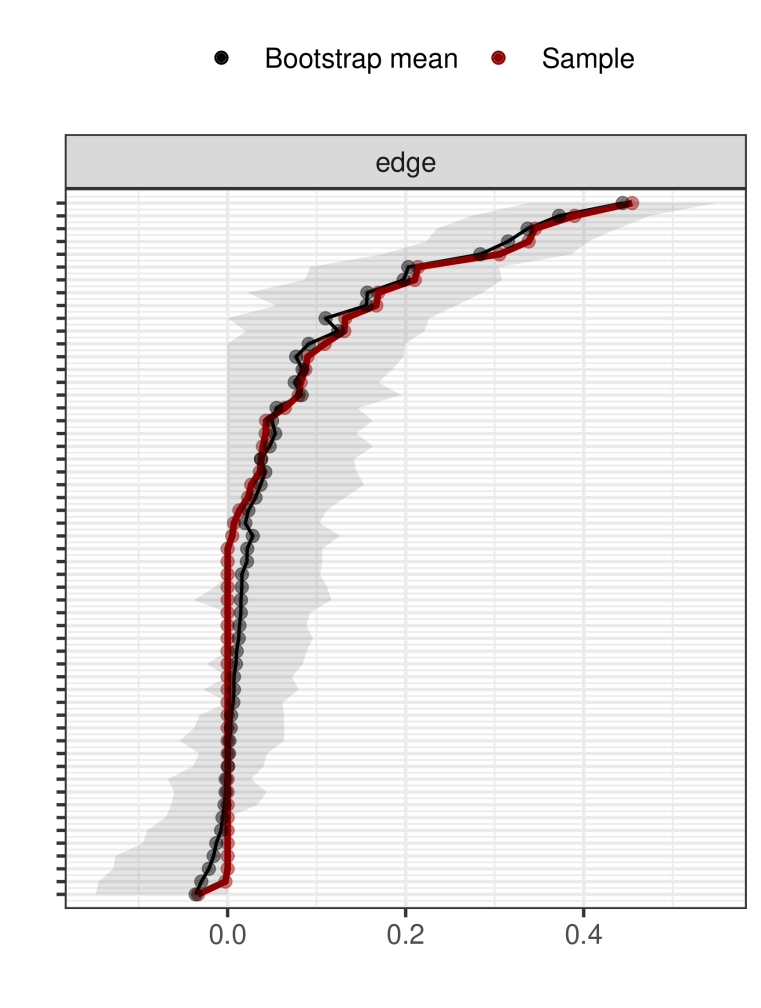


Case-dropping procedure to evaluate centrality stability.

Edge-weights are sorted in increasing order (red). Grey areas represent 95% confidence intervals. Edge-edge relationships are depicted on the y- axis with labels.

**Supplementary figure 20:** Centrality difference test of expected influence (left) and strength (right).


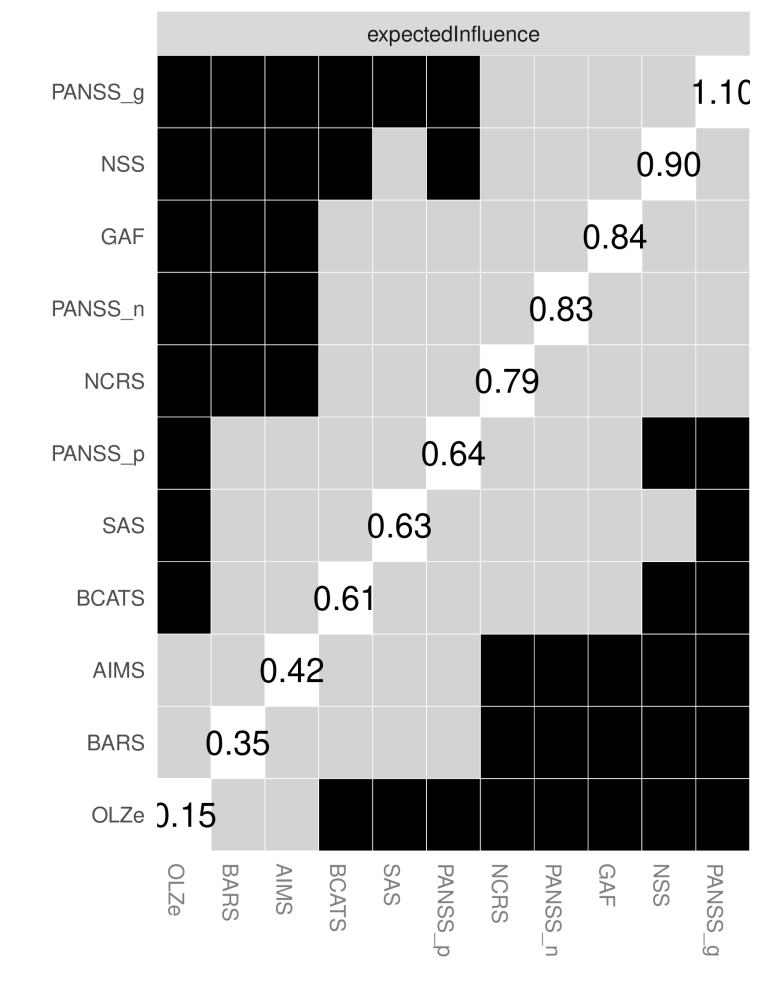

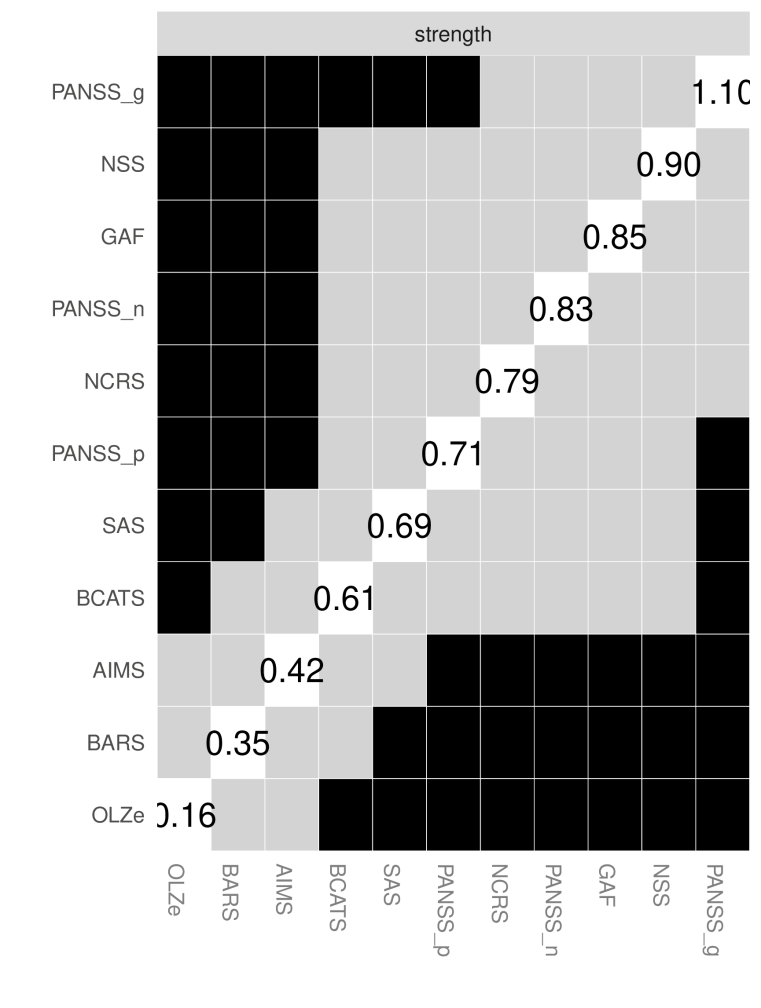


Black tiles represent significantly different centralities. Grey tiles represent non-significantly different centralities.

**Supplementary figure 21:** Edge-weight difference test.


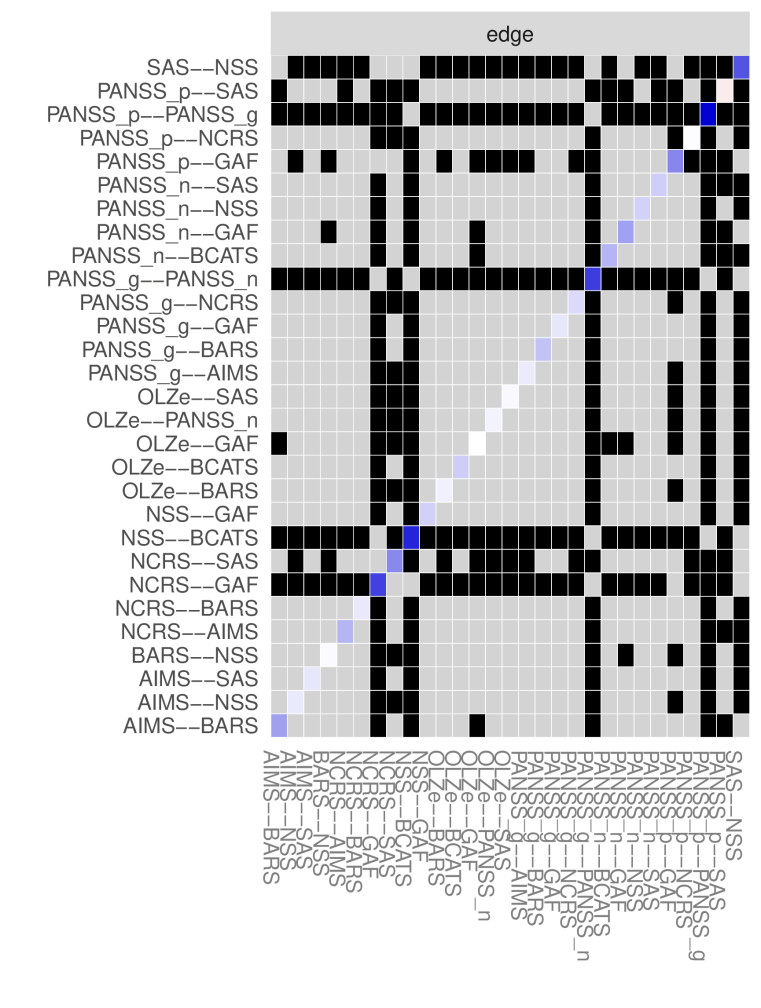


Black tiles represent significantly different edge-weights. Grey tiles represent non-significantly different edge-weights.
